# DNA methylation of the glucocorticoid receptor gene predicts substance use in adolescence: longitudinal data from over 1000 young individuals

**DOI:** 10.1038/s41398-021-01601-6

**Published:** 2021-09-15

**Authors:** Elena Raffetti, Philippe Anastasios Melas, Anton Jonatan Landgren, Filip Andersson, Yvonne Forsell, Catharina Lavebratt, Maria Rosaria Galanti

**Affiliations:** 1 Department of Global Public Health, Karolinska Institutet, Stockholm, Sweden; 2 Center for Psychiatry Research, Department of Clinical Neuroscience, Karolinska Institutet & Stockholm Health Care Services, Stockholm, Sweden; 3 Center for Molecular Medicine, Karolinska University Hospital, Stockholm, Sweden; 4 Region Västra Götaland, Research and Development Primary Health Care, Gothenburg, Sweden; 5 Department of Rheumatology and Inflammation Research, Institute of Medicine, Sahlgrenska Academy, University of Gothenburg, Gothenburg, Sweden; 6 Centre for Epidemiology and Community Medicine, Stockholm Health Care District, Stockholm Region, Stockholm, Sweden; 7 Department of Molecular Medicine and Surgery, Karolinska Institutet, Stockholm, Sweden

**Keywords:** Predictive markers, Epigenetics and behaviour

## Abstract

Early life stress has been linked to increased methylation of the Nuclear Receptor Subfamily 3 Group C Member 1 (*NR3C1*) gene, which codes for the glucocorticoid receptor. Moreover, early life stress has been associated with substance use initiation at a younger age, a risk factor for developing substance use disorders. However, no studies to date have investigated whether *NR3C1* methylation can predict substance use in young individuals. This study included adolescents 13–14 years of age that reported no history of substance use at baseline, (*N* = 1041; males = 46%). Participants contributed saliva DNA samples and were followed in middle adolescence as part of KUPOL, a prospective cohort study of 7th-grade students in Sweden. Outcome variables were self-reports of (i) recent use, (ii) lifetime use, and (iii) use duration of (a) alcohol, (b) tobacco products, (c) cannabis, or (d) any substance. Outcomes were measured annually for three consecutive years. The predictor variable was DNA methylation at the exon 1 F locus of *NR3C1*. Risk and rate ratios were calculated as measures of association, with or without adjustment for internalizing symptoms and parental psychiatric disorders. For a subset of individuals (*N* = 320), there were also morning and afternoon salivary cortisol measurements available that were analyzed in relation to *NR3C1* methylation levels. Baseline *NR3C1* hypermethylation associated with future self-reports of recent use and use duration of any substance, before and after adjustment for potential confounders. The overall estimates were attenuated when considering lifetime use. Sex-stratified analyses revealed the strongest association for cigarette use in males. Cortisol analyses revealed associations between *NR3C1* methylation and morning cortisol levels. Findings from this study suggest that saliva *NR3C1* hypermethylation can predict substance use in middle adolescence. Additional longitudinal studies are warranted to confirm these findings.

## Introduction

Adolescence represents a critical period for brain maturation and development that, if disrupted by substance use, can enhance the risk for developing substance use disorders later in life [1]. Individuals who initiate substance use before the age of 14 have an estimated 34% prevalence rate of lifetime abuse and, as individuals continue to mature until the age of 21, the risk drops 4–5% for each year that substance use initiation is delayed [2, 3]. Exposure to stressful life events in childhood is a strong risk factor for a younger age of drug use onset and the emergence of problematic substance use already in adolescence [4–8]. However, the molecular mechanisms that underlie the association between early life stress and adolescent substance use remain largely unknown.

Stress activates the hypothalamic–pituitary–adrenal (HPA) axis and leads to the production of glucocorticoids, e.g., cortisol in humans [9]. Glucocorticoids serve numerous functions essential to survival, including metabolic and inflammatory processes, and they mediate the behavioral responses to stress [9]. Glucocorticoids also act in a negative feedback loop to suppress HPA activation and promote homeostasis when the stressor has subsided [9]. On the molecular level, this negative feedback is achieved by binding of glucocorticoids to glucocorticoid receptors, transcription factors encoded by the Nuclear Receptor Subfamily 3 Group C Member 1 (*NR3C1*) gene [9]. Preclinical rodent models of early life stress, e.g., based on maternal separation, have linked prolonged activations of the HPA axis with the development of anxiogenic and fearful behaviors [10]. The negative effects of maternal separation were found to be mediated by epigenetic changes in the rodent *Nr3c1* gene. Specifically, a relationship was found between maternal separation and DNA hypermethylation of the neuron-specific *Nr3c1* promoter, at the exon 1_7_ locus, which interferes with transcription factor binding of the nerve growth factor-induced protein A (NGFI-A) and leads to decreased expression of *Nr3c1* [11]. A number of human studies have also found that *NR3C1* hypermethylation at the equivalent human locus (i.e., exon 1 F) is associated with exposure to early life stress, including childhood maltreatment, parental loss, or parental disease, and being the victim of bullying [12–19]. Moreover, several human studies have linked *NR3C1* methylation levels with changes in HPA functioning, e.g., as reflected by aberrant cortisol stress responses [15, 17, 20–22].

There is also increasing evidence demonstrating that an altered HPA axis, e.g., due to early life adversity, can contribute to key aspects of substance use disorders [23, 24]. Specifically, a dysregulated HPA axis can enhance a drug’s positive reinforcing effects, thus promoting continued use that is necessary for the development of dependence [25]. A dysregulated HPA axis can also strengthen the negative effects associated with abstinence and withdrawal, thus contributing to relapse and the maintenance of substance use through negative reinforcement [26, 27]. Glucocorticoids, in particular, interact with various neurotransmitter and neuropeptide systems implicated in substance use disorders and are thought to play a critical role in addiction, partly by modulating the formation of drug-related memories [28, 29]. A specific and causal role of NR3C1 in addictive behaviors has been demonstrated in preclinical studies, where selective inactivation of *Nr3c1* in mouse dopaminoceptive neurons was found to reduce the motivation of mice to self-administer cocaine [30]. Similarly, pharmacological compounds targeting NR3C1, including NR3C1 antagonists and modulators, have been found to possess therapeutic properties in intervention studies for alcohol dependence [31, 32].

However, no large-scale human studies to date have employed a longitudinal approach to examine the association between *NR3C1* methylation at the exon 1 F locus and later substance use risk among adolescent individuals. The presented data, which derive from over 1000 adolescents with no reported history of substance use at baseline (i.e., at 13–14 years of age), suggest that saliva *NR3C1* hypermethylation can predict future substance use in middle adolescence.

## Methods

### Study participants

The study participants were part of the KUPOL study, a prospective cohort study that included *N* = 3959 adolescents attending the 7th grade (13–14 years of age) of compulsory school in both urban and rural areas of eight regions in southern and central Sweden. Detailed information on the KUPOL study has been published elsewhere [33]. In brief, participant recruitment took place during school years 2013–2014 and 2014–2015. Data were collected through self-administered questionnaires that were completed annually by adolescents and their parents during the following three years, i.e., until 16–17 years of age. National registries were also used to retrieve medical and socio-economic information on students and their parents. A sample of KUPOL students also contributed saliva specimens (*N* = 1315). In the present study, we analyzed participants who were naïve to any substance use at baseline and had successful measurements of *NR3C1* methylation (*N* = 1041). The KUPOL study was approved by the Stockholm Ethics Review Board (reference numbers: 2012/1904-31/1 and 2016/1280-32). All students agreed to participate in the study and all legal guardians gave written informed consent.

### Substance use data

Self-reported substance use data were available for cigarettes, snus (a smokeless tobacco product popular in Scandinavia), alcohol, and cannabis. In the analyses, we also included the compound substance measures of “tobacco use” and “any substance use”. “Tobacco use” was defined as cigarette smoking and/or snus use, and “any substance use” was defined as any report of using tobacco, alcohol, or cannabis. For each substance, we analyzed three main outcomes, i.e., (i) recent use, (ii) lifetime use, and (iii) use duration, according to the following criteria: (i) Recent tobacco or cannabis use was defined as a report of first-time use in the past 30 days [yes/no]. Recent alcohol use was defined as a first-time report of having consumed alcohol at least once a month during the past year. (ii) Lifetime use was defined as the first-time report of having used tobacco or cannabis at least once in a lifetime (i.e., also capturing use prior to the past 30 days) or having consumed alcohol at least once in the past year (i.e., also capturing the use of less than once a month in the past year) [yes/no]. Since none of the individuals had used any substances at baseline, lifetime use referred to the entire lifetime of the individuals up to the examined point. (iii) Use duration of each substance was calculated as the number of years reporting recent use in the 3-year follow-up (i.e., range between 0 and 3).

### DNA methylation analysis

**Fig. 1:**
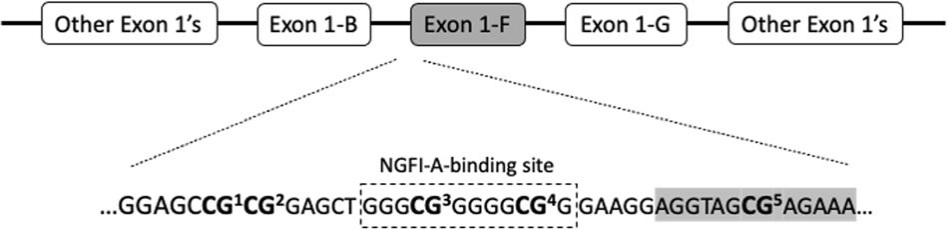
The structure of *NR3C1* exon 1 variants. The figure shows the *NR3C1* gene structure of exon 1 variants, as reported by McGowan et al. [13]. Below exon 1-F, we show part of the promoter and exon 1-F nucleotide sequences, with the five CpG sites analyzed in our study, denoted in bold (CG^1^, CG^2^, CG^3^, CG^4^, and CG^5^). Our CG^1^-CG^5^ sites correspond to CG^35^–CG^39^ sites analyzed in McGowan et al. [13]. The nucleotide sequence inside the broken box corresponds to a binding site of the nerve growth factor-induced protein A (NGFI-A) in the proximal promoter region of exon 1-F, as reported by McGowan et al. [13]. The gray-shaded area of the exon 1-F sequence indicates the beginning of the exon.

Detailed procedures of DNA sample preparation and DNA methylation analyses have been described previously [34]. In brief, saliva samples were collected from participants using whole-saliva collection kits (Oragene•DNA; DNA Genotek Inc., Ottawa, Canada) and, following DNA extraction, bisulfite conversion of the DNA was performed using the EZ-96 DNA Methylation-Gold^™^ MagPrep kit (Zymo Research Corporation; Irvine; USA). DNA methylation levels at the *NR3C1* exon 1-F locus were quantified using pyrosequencing reagents on a Pyromark Q96 device (Qiagen, Hilden, Germany). Specifically, a 162-base pair fragment was amplified using the PyroMark PCR Kit (Qiagen, Hilden, Germany) and the following primers: forward 5′- AGTTTTAGAGTGGGTTTGGAG-3′, reverse biotin-5′-CCCCCAACTCCCCAAAAA-3′. Polymerase chain reaction conditions were optimized to yield distinct single bands and were as follows: (i) 94 °C for 15 min, (ii) 45 cycles of 94 °C for 30 s, 60 °C for 60 s and 72 °C for 30 s, and (iii) final extension at 72 °C for 10 min. The sequencing primer, 5′-GAGTGGGTTTGGAGT-3′, was used for pyrosequencing, and data were collected on five CpG sites, denoted CpGs 1–5 (Fig. 1). As shown in Fig. 1, CpGs 3 and 4 belong to a binding site of NGFI-A, as reported by McGowan et al. [13]. Our CpG numbering follows the one reported by Oberlander et al. [17], where CpG3 was also found to associate with infant stress cortisol reactivity. Of note, CpG3 constitutes a site that is not captured by the HumanMethylation450 and the EPIC BeadChips (Illumina; Illumina Inc., San Diego, CA, USA). For reference, we also provide the 450 K/EPIC array probe IDs that correspond to our remaining CpGs of interest: CpG1 (cg15645634), CpG2 (not captured), CpG3 (not captured), CpG4 (cg15910486), CpG5 (cg04111177). To validate the efficiency of our primers, amplification of unmethylated and methylated control DNA samples was performed using human HCT116 DKO Non-Methylated and Methylated DNA (Zymo Research Corporation). The percentage methylation values obtained for the unmethylated control was in the 0–4% range, and for the fully methylated control in the 86–95% range, for all five CpGs. Water samples gave no signal. The average between-plate coefficient of variation for CpGs 1–5 was 10.3% (range: 4.5–16.2%), calculated from control samples containing 0, 6, 9, and 12% methylated DNA, analyzed in duplicate in each plate. Most of the analyzed samples (98%) yielded methylation values within a 0–12% range at any given CpG site, which is within the commonly reported range (0–20%) described in previous pyrosequencing studies of the same NR3C1 region using blood or saliva samples from infants, children, and adults [15, 17, 22, 35]. In our study, correlation analyses among the five CpG sites also showed that CpG3 was the site whose DNA methylation levels correlated the least with the remaining four sites (Fig. S1). We also provide the overall distribution of methylation levels across the five CpG sites (Fig. S2), as well as the distributions of individual CpG methylation levels in relation (i) to sex, internalizing symptoms, and parental history of psychiatric disorders (Fig. S3) and (ii) to substance use types (Fig. S4). All our analyses were performed blinded to the phenotypes and, for analytical purposes, methylation levels for each CpG were categorized into three groups as previously described [34], i.e.,: (i) unmethylated: participants with no detectable methylation levels (0%); (ii) low methylation: methylation below the median of detectable methylation levels; and (iii) high methylation: methylation above the median of detectable methylation levels.

### Salivary cortisol analyses

Salivary cortisol data were available from 320 participants at baseline. The specimen collection and analytic procedures for salivary cortisol measurements have been described previously [36]. In brief, specimens were collected in Salivette tubes (Sarstedt, Leicester, UK) twice during the same day; in the morning (approximately 2 h after awakening) and in the afternoon (approximately 8 h after awakening). Samples were stored at −20 °C until subsequent cortisol measurements using an enzyme-linked immune sorbent assay (Salivary Cortisol ELISA Kit; Salimetrics, UK). Each ELISA sample was run in duplicate, with morning and afternoon samples from the same participant run on the same 96-well plate, and cortisol concentrations were expressed in micrograms per deciliter (μg/dL). Each plate also contained a cortisol concentration standard curve, as well as interplate controls with known cortisol concentrations, run in duplicates. The correlation coefficients of the standard curves were >0.997 with a median of 0.999 for each plate. The control samples revealed a between-plate coefficient of variation of 7.0% and the median within-plate coefficient of variation for all participant samples was 8.1%.

### Covariates

We used information on parents’ education, birthplace, and smoking habits to examine the social characteristics of the sample. Previous literature has found that methylation of *NR3C1* at the exon 1-F locus is associated with internalizing, but not externalizing, behavior problems [32, 34]. Moreover, parental depression has been associated with *NR3C1* methylation in the offspring [17]. Thus, adolescent internalizing symptoms and parental psychiatric disorders were considered potential confounders. However, it is also important to consider another possible causal pathway. Specifically, since internalizing symptoms can stem from a dysregulated HPA axis, internalizing symptoms may be a mediator of the association between HPA-axis dysregulation and substance use. Thus, considering internalizing symptoms as a covariate in the models may result in over adjustment. We included self-reported internalizing symptoms (as a continuous variable) in the 7th grade, measured according to the Center for Epidemiologic Studies Depression Scale for Children (CES-DC) [37, 38]. The CES-DC is a 20-item, internationally validated scale used in epidemiological studies of children and adolescents (6–17 years of age), with a total score ranging from zero to 60. This score was dichotomized using a cut-off score of ≥30, which has been found to capture internalizing problems in Swedish adolescents [38, 39]. Parental history of mental, behavioral, and neurodevelopmental disorders was retrieved from the National Patient Register according to the International Classification of Disease (ICD) 10 codes (F01-F99) [40] and categorized as a binary variable.

### Statistical analyses

We used descriptive statistics to summarize the main characteristics of the study sample. The associations of *NR3C1* methylation with recent substance use, lifetime substance use, and substance use duration were assessed on a univariate level for each CpG site separately and for all sites grouped together. Poisson regression models $\left( {\log \left( {E\left( {Y{{{\mathrm{|}}}}X} \right)} \right) = \alpha + \beta _i \ast X_i} \right)$, with or without sex stratification, were used to derive the rate ratios (RRs) and corresponding 95% confidence intervals (CIs) for recent use and lifetime use, and the risk ratios (RRs) and 95% CIs for substance use duration and increasing methylation during the 3-year follow-up. In all analyses, the unmethylated group constituted the reference. Models with and without adjusting for internalizing symptoms and parental history of mental, behavioral, and neurodevelopment disorders were compared. A sensitivity analysis was performed to analyze the potential selection bias introduced by attrition in the third follow-up (non-compulsory education, 18.6%), limiting the analyses to the first two years of follow-up (end of the Swedish compulsory education). In addition, multilevel models were used to take into account the clustering of students within schools. As multilevel models did not reveal heterogeneity among schools, the results were reported according to the standard regression models. Bonferroni-corrected confidence intervals were included in sensitivity analyses to adjust for multiple testing. Confidence intervals were set at 99.2% (1-alpha/*m*, where alpha = 0.05 [type I error] and *m* = 6 [number of tests, 5 separate CpG sites + CpG sites grouped together]). The goodness of fit test and graphical checks of residuals based on residual deviance did not show a major departure from Poisson model assumptions.

To quantify the effect of unmeasured potential confounding factors, we also report the *E*-values for associations between CpG 3 site/CpG 1–5 sites and substance use as outcomes. The *E*-value represents the minimal strength of association on the RR scale that an unmeasured confounder would need to have, with both the predictor and the outcome, to fully explain the association between the two [41].

The association between the methylation levels across the five CpG sites and cortisol levels were examined using linear regression models. Due to the small sample size (*n* = 320), methylation levels were considered as a binary variable (methylated vs. unmethylated group). To account for potential selection because of the sampling approach, we performed weighted linear regression models applying an inverse probability weight method. Sex, internalizing problems, parental history of mental, behavioral, and neurodevelopmental disorders, and school was considered in weight calculation. As the results from weighted models were not different from those obtained with standard linear regression models, they are reported according to this latter method.

The code of the statistical analysis is available from the corresponding author on request.

Considering a prevalence of methylation of 50% for CpG 3 site, a sample of 1040 students is sufficient to detect a 5% increase in smoking behaviors in those methylated compared to those non-methylated with a power of 80%.

The Stata software package (StataCorp. LLC; TX, USA) was used for all statistical analyses.

## Results

### Participant characteristics

The analytical sample consisted of *N* = 1041 students, 13–14 years of age, without a history of substance use at baseline. Of these students, 73.5% had at least one parent with a university education, less than 20% had an immigration background with similar distribution between sexes (Table 1). Also, 6.2% of students presented with internalizing symptoms according to CES-DC scoring, with a higher prevalence in females than in males (9.3% vs. 2.3%), and 11.5% of students had parents with a history of psychiatric disorders. The prevalence of methylation varied across the five analyzed CpG sites of *NR3C1* and CpG3, which belongs to the binding site of NGFI-A in the promoter region of *NR3C1*’s exon 1-F (Fig. 1), was the site most prone to methylation (Table 1). The distribution of methylation levels was similar according to sex and internalizing symptoms distribution, while the parental history of psychiatric disorders was associated with higher levels of methylation at CpG sites 1–3 (Fig. S3).

**Table 1:** Characteristics of the study population at baseline (13–14 years of age).

		Total (*n* = 1041) *n* (%)	Males (*n* = 476) *n* (%)	Females (*n* = 565) *n* (%)
At least one parent with university education	Yes	760 (73.5)	349 (73.8)	411 (73.3)
At least one parent born abroad	Yes	178 (17.9)	72 (15.9)	106 (19.5)
Saliva samples with methylation >0% for each CpG site	CpG1	182 (17.5)	78 (16.4)	104 (18.4)
	CpG2	130 (12.5)	59 (12.4)	71 (12.6)
	CpG3	526 (50.5)	243 (51.1)	283 (50.1)
	CpG4	199 (19.1)	97 (20.4)	102 (18.1)
	CpG5	88 (8.5)	36 (7.6)	52 (9.2)
	CpGs 1–5^a^	637 (61.2)	294 (61.8)	343 (60.7)

^a^Methylation > 0% in any CpG site.

During the 3-year follow-up, 166 students (15.9%) reported recent use of at least one substance. Specifically, 96 students (9.2%) reported recent smoking, 56 (5.4%) recent use of snus, 103 (9.8%) recent use of alcohol, and 14 (1.3%) recent use of cannabis. Totally, 76 (7.3%) students reported recent polysubstance use (Table S1). We observed slightly higher recent use rates among females than males (18.0% versus 13.5% for at least one substance) particularly for cigarette smoking (11.3% versus 6.7%) and alcohol consumption (12.2% versus 7.1%). With regard to lifetime use, 193 students (18.5%) reported ever having smoked, 118 (11.3%) reported lifetime use of snus, 486 (46.7%) reported lifetime use of alcohol, and 35 (3.4%) reported lifetime use of cannabis (Table S1). With regard to substance use duration, about 10% of students used at least one substance for 1 year, 5% for 2 years, and 1% for 3 years (Table S1). Table S2 reports descriptive statistics on the association between DNA methylation levels and current substance use. The groups with high methylation levels for CpGs 2 and 3, and CpGs1–5, show higher current substance use. The graphical visualization of the distribution of methylation levels confirmed this pattern, mainly for CpG site 3 (Fig. S4).

### NR3C1 methylation predicts substance use in adolescence

**Fig. 2:**
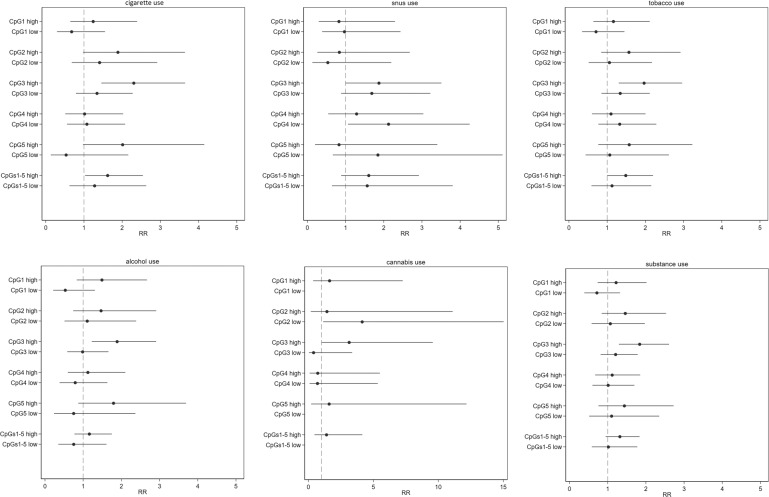
Bi-variate association between NR3C1 methylation at baseline and self-reports of recent use assessed over 3 years. The results should be interpreted in terms of rate ratios (RRs) with 1 as a null value. Methylation level 0% was considered as the reference category. Low methylation group: methylation levels below the median of detectable methylation and high methylation group: methylation levels above the median. RRs were not estimated for the CpG1 CpG5 and CpG1–5 sites with low methylation for cannabis use given no cases in these groups. The cases/total are available in Table S2.

The results from the Poisson regression models that compared rates of recent substance use, in terms of baseline *NR3C1* methylation levels, are presented in Fig. 2 and Table S3. Students with high baseline DNA methylation levels at CpG site 3 had 1.7–2.3-fold higher rates of reporting recent use of cigarettes, snus, and alcohol. The estimates across the other CpG sites (2, 4, and grouped) were lower, although the same patterns emerged, especially for the recent use of cigarettes. Thus, high baseline methylation in the combined CpG1–5 group is associated with an increased risk for recent cigarette use. The sensitivity analysis showed that the corresponding *E*-value for methylation at CpG site 3 (RR = 2.30) and any CpG site (RR = 1.62) as exposure, and recent cigarette use as an outcome, were 4.03 (lower confidence limit 2.26) and 2.62 (1.21), respectively (Table S4). The multivariable-adjusted analysis for internalizing symptoms and parental history of psychiatric disorders, along with the Bonferroni-corrected estimates, yielded similar results but with less precise estimates (Tables S5 and S6). The only exception was for cannabis use where the confidence intervals were too wide to draw any conclusion on the direction of the association.

Site- and substance-specific associations were slightly different between sexes (Tables S7 and S8). Risk estimates were stronger for CpG site 3 in males, and CpG site 2 in females. Moreover, the associations with cigarette and alcohol use were more pronounced in males.

**Fig. 3:**
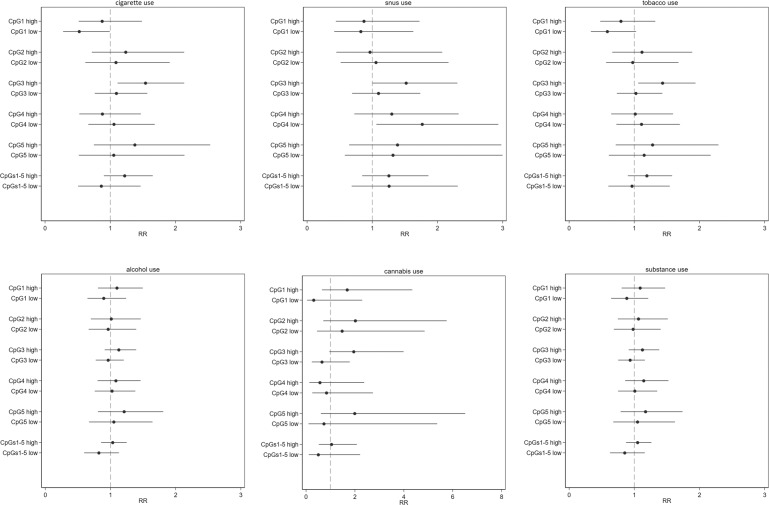
Bi-variate association between NR3C1 methylation at baseline and lifetime substance use assessed over 3 years in adolescent participants of the KUPOL study. The results should be interpreted in terms of rate ratios (RRs) with 1 as a null value. Methylation level 0% was considered as the reference category. Low methylation group: methylation levels below the median of detectable methylation and high methylation group: methylation levels above the median.

The overall estimates were attenuated when considering lifetime substance use as the outcome (Fig. 3 and Table S9) also considering models adjusted for parental history of psychiatric disorders and with Bonferroni corrected estimates (Tables S10 and S11). Nonetheless, the associations remained significant between CpG site 3 and cigarette smoking or cannabis use. Moreover, sex-stratified analyses revealed associations for lifetime cigarette use in males (CpG site 3) and lifetime cannabis use in females (CpG sites 1 and 5) (Tables S12 and S13).

### NR3C1 methylation predicts substance use duration in adolescence

**Fig. 4:**
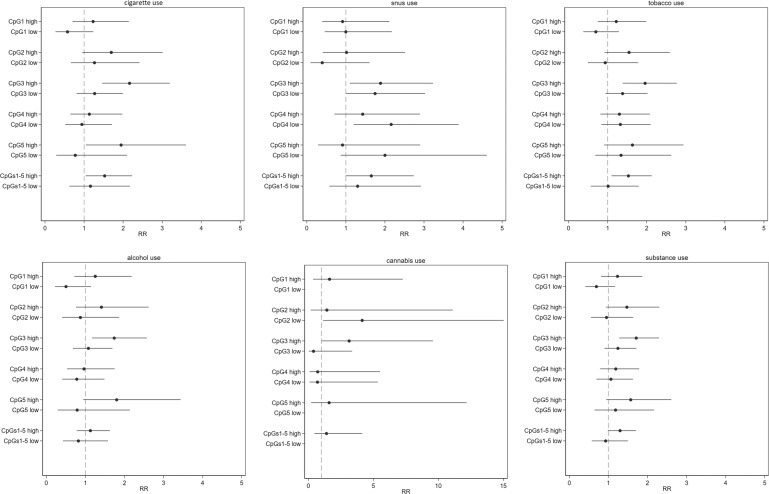
Bi-variate association between NR3C1 methylation levels at baseline and duration of substance use (per year increase) assessed over three years in adolescent participants of the KUPOL study. The results should be interpreted in terms of risk ratio (RRs) with 1 as a null value. Methylation level 0% was considered as the reference category. Low methylation group: methylation levels below the median of detectable methylation and high methylation group: methylation levels above the median. RRs were not estimated for the CpG1 CpG5 and CpG1–5 sites with low methylation for cannabis use given no cases in these groups.

Baseline *NR3C1* methylation levels were also associated with higher risk for prolonged substance use duration (i.e., number of years reporting recent use), with the highest estimates in those with high methylation levels at CpG site 3 in both unadjusted and adjusted models (Fig. 4, Tables S14–S16). Site- and substance-specific associations were also confirmed for the duration outcome when stratifying by sex (Tables S17 and S18). Restricting analyses to the second follow-up resulted in estimates that were in the same direction but with wider confidence intervals (data not shown).

### Association between NR3C1 methylation and salivary cortisol levels

Finally, we examined the association of *NR3C1* methylation at the five CpG sites under investigation with morning or afternoon salivary cortisol levels, which were available from a subset of participants (*N* = 320). We found that DNA methylation levels at CpG site 3 were associated with higher morning cortisol levels. The average increase of morning cortisol level was 0.027 μg/dL [95% CI: 0.003–0.050] in the methylation group compared to the unmethylated group. No significant associations were found for the remaining CpG sites or with afternoon cortisol levels.

## Discussion

In this prospective cohort study of over 1000 adolescent individuals, we found that the presence of hypermethylation at the exon 1-F promoter of *NR3C1* in early adolescence, i.e., 13–14 years of age, was associated with an increased risk of future self-reports of recent substance use and substance use duration during the 3-year follow-up. When considering lifetime substance use as an outcome, the overall estimates were attenuated although some significances remained. Specifically, our findings were particularly consistent for hypermethylation of CpG site 3, which belongs to a binding site of NGFI-A and has been associated with decreased *NR3C1* expression as part of the exon 1-F promoter region [13]. In our study, hypermethylation of CpG site 3 predicted future self-reports of (i) recent use of any substance (i.e., tobacco products, alcohol, or cannabis), (ii) lifetime use of tobacco products (cigarettes or snus), as well as (iii) substance use duration of any substance. Sex-stratified analyses revealed a pronounced association in males between CpG3 hypermethylation and recent use of cigarettes (RR = 5.3). The equivalent analyses in females revealed an association between hypermethylation of CpG2 and recent tobacco use (RR = 2.31). However, hypermethylation of CpG3 was associated again with a longer duration of tobacco use in both males and females. Adjusting for potential confounders, such as internalizing problems and parental history of mental, behavioral, and neurodevelopmental disorders, yielded results in the same direction.

Lifetime substance use can be viewed as a proxy for risk-taking behavior often observed in adolescence, and with predisposing factors that include temperament, neurobehavioral disinhibition, and peer behaviors [42]. As such, “lifetime use” can be considered a more benign outcome since it also captures individuals who are occasional users or have experimented with a substance on single occasions. By contrast, the transition from occasional use to a substance use disorder constitutes a maladaptive process that requires both heavy/repeated use and genetic predispositions [43]. Thus, the frequency of recent substance use reports over the years (reflected in our study using the outcome of substance use duration), may be a better indicator of chronic problematic substance use, which increases the risk of substance use disorders [42, 44]. Given (i) the overall attenuated estimates for lifetime use found in our study, and given (ii) that methylation quantitative trait loci (mQTLs) may underlie our observed changes in *NR3C1* methylation (as discussed further below), this may indicate that *NR3C1* is involved more in those maladaptive processes that are related to substance abuse, and less in those processes that underlie experimental or occasional use. However, further longitudinal studies, with more detailed information on substance use behaviors, are warranted to support this hypothesis.

The first evidence that *NR3C1* may be able to modulate the initiation of substance use, was provided by genetic studies showing a significant association between polymorphisms in the *NR3C1* gene and alcohol use initiation in 14‐year‐old adolescents [45]. However, our study is the first to our knowledge to employ a large-scale (*N* > 1000) longitudinal approach to examine whether *NR3C1* methylation at the exon 1-F locus can predict three different substance use outcomes in middle adolescence. Importantly, the use of DNA samples from individuals with no lifetime history of substance use, makes our findings unlikely to be explained by reverse causality [46, 47]. Moreover, since previous studies had found associations between *NR3C1* methylation and cortisol stress responses [15, 17, 20–22], we also examined the correlations between the five CpGs under investigation and salivary cortisol levels available from a subset of individuals. We found that DNA methylation at CpG 3 was associated with higher morning cortisol levels. Interestingly, a previous study using cord blood samples from infants found that increased methylation at CpG3 was the only CpG site under investigation that was associated with infant stress cortisol reactivity [17]. The same study found that exposure to increased third-trimester depressed maternal mood was also associated with increased neonatal methylation of CpG3 [17]. Similarly, an independent study using preschoolers found that hypermethylation at the same CpG site is associated with internalizing behavior problems [35], providing further support for the biological relevance of this CpG site and its association to stress sensitivity, HPA functioning, and risk for psychopathology.

Overall, our data suggest that saliva *NR3C1* hypermethylation can predict substance use outcomes in middle adolescence, and are consistent with our previous findings from the KUPOL study, which demonstrated an association between morning cortisol levels and tobacco use initiation [36]. Moreover, our findings provide support for an epigenetic predisposition that could drive vulnerability to addiction [48]. Although no prior substance-use studies employed a longitudinal approach to investigate *NR3C1* methylation levels in adolescents, a number of studies used cross-sectional approaches in adult cohorts, which provide support for our findings. Specifically, two studies investigating alcohol use found hypermethylation and lower expression of *NR3C1* in post-mortem brain tissue of adult individuals with a history of alcohol use disorder [49, 50]. Another study investigating cocaine use found lower peripheral levels of *NR3C1* gene expression in adult chronic cocaine users [51]. A study using healthy adults found that a history of a *past* substance use disorder was associated with lower peripheral levels of *NR3C1* methylation [52]. However, a recent study that examined adults with cannabinoid use disorders found no associations with *NR3C1* methylation levels [53].

The specific contributors to *NR3C1* hypermethylation in our study participants remain unknown. Although previous studies have provided support for the hypothesis that *NR3C1* hypermethylation in children can result from environmental adversities, such as early life stress [54], we were not able to address this possibility since our utilized cohort did not include information on early life adversities. Moreover, we cannot exclude the possibility that genetic variations can also influence *NR3C1* methylation levels, similar to findings from other stress-responsive genes such as the FK506 binding protein 5 (*FKBP5*) gene [55, 56]. Moreover, it is unlikely that methylation of *NR3C1* is the only epigenetic predisposing factor influencing substance use risk in adolescence. This assumption is supported by the only additional prospective study of DNA methylation and substance use risk in adolescence, to our knowledge, which included 244 individuals with methylation data from cord blood at birth and whole blood at age 7 [57]. This study by Cecil and colleagues found no link with *NR3C1* but reported instead of an association between substance use during adolescence and cord blood methylation of 65 loci involved in neurodevelopmental processes, including *PACSIN1*, *NEUROD4*, and *NTRK* [57]. Nonetheless, it should be noted that the latter study was conducted using the Illumina HumanMethylation450 BeadChip, which does not capture our main CpG site of interest, i.e., CpG3 (see also Methods). Moreover, although Illumina and pyrosequencing data have been found to be congruent to a large extent, deviations from this congruency may still occur and caution needs to be taken for individual loci when translating beta-values directly into percent methylation levels [58].

A number of additional limitations should be kept in mind when interpreting the results from our study. First, the level of DNA methylation was evaluated at a single time point, making the timing of the epigenetic process unclear. Second, DNA methylation levels detected in saliva do not necessarily correspond to those in the brain since DNA methylation signatures are often tissue-specific. Nonetheless, previous studies examining the concordance of DNA methylation across commonly used peripheral tissues (i.e., blood, saliva, and buccal tissue) with DNA methylation in the brain, found the highest correlations to the brain with saliva, at least when the average global correlations were used [59]. Thus, it is also important to consider the nature of the cell types present in saliva samples, which may vary based on each study’s saliva collection kit. For instance, the epithelial cell count in saliva samples collected with the Oragene·DNA kit, used in our study, can range from 20 to 70%, with the remaining cells being mostly leukocytes and with the age of subject being one potential determining factor (personal correspondence with DNA Genotek). Furthermore, although it is customary to assess leukocyte cell-type distributions in blood-based DNA methylation studies, such cell-type assessments are not made possible in saliva samples already collected with the Oragene·DNA kit that utilizes lytic chemistry, meaning it will lyse most cells in the sample and degrade/denature proteins. To address this limitation, future studies can consider collecting a small amount of saliva into a separate tube for cell sorting, since raw saliva samples can be used to perform cell-sorting assays and can be smeared on a slide to enumerate cells by microscopy. Nonetheless, it is reassuring that the majority of our analyzed samples yielded methylation values within the commonly reported range described in previous pyrosequencing studies of *NR3C1* using blood or saliva samples [15, 17, 22, 35].

Third, the number of participants with drug use was relatively small, which could affect the precision of the results. In addition, self-reported substance use tends to underestimate actual use [60, 61]. Fourth, randomness in methylation can affect measurement precision, while systematic measurement errors may bias estimates towards or away from the null. Nonetheless, the large sample size contributed to reducing the role of random errors on final estimates. However, the small number of cases for cannabis use did not always allow reaching conclusions on estimated directions. Moreover, possible measurement errors of methylation are unlikely to have occurred differentially according to substance use. Hence, nondifferential misclassification would likely result in an underestimation of the associations under examination. Along the same lines, misclassification of substance use is unlikely to have differed according to methylation levels. Nonetheless, caution needs to be taken when interpreting results from substance use duration, since a value of 1 at the first follow-up may differ in meaning from a value of 1 at the third follow-up. Specifically, the former may indicate experimental use if it is not followed by substance use in the second follow-up, while the latter may indicate either experimental use or the beginning of chronic use depending on substance intake during the subsequent (unstudied) years. Finally, unmeasured, unknown, or residual confounding could not be excluded, for instance, due to parenting style and genetic liability to substance dependence. Consequently, we performed a sensitivity analysis to estimate the magnitude of confounding (Table S4). For example, the observed RR of 1.62 (between grouped CpG sites and recent cigarette use) could be explained by an unmeasured confounder that was associated both with the exposure and the outcome by a RR of 2.62 each (*E*-value), above and beyond the measured confounders. The confidence interval could be moved to include the null by an unmeasured confounder that was associated both with the exposure and with the outcome by a RR of 1.21 each, above and beyond the measured confounders. Overall, *E*-values support the robustness of the present findings. Indeed, genetic factors in the form of single nucleotide polymorphisms (SNPs) represent the main possible confounders in the pathway between HPA axis dysregulation and substance use. However, it is unlikely that the magnitude of the association between a single SNP and both exposure/outcome will be higher than 2.62, given that psychiatric traits are most polygenic, involving a continuum of small effects, and SNP heritability accounts for modest percentages in substance use behaviors [43, 62, 63].

Despite these limitations, our study also had notable strengths. It was based on a large longitudinal cohort with low attrition at first and second follow-ups (8.9% and 12.7%, respectively), thus minimizing the risk of reverse causality due to DNA methylation changes caused by substance use [64]. DNA methylation was assessed in early adolescence, a period when the use of substances is usually not yet established, and the few subjects that reported substance use at baseline were excluded. Moreover, outcome information was assessed during three consecutive years. The adjustment was also made for internalizing symptoms and parental history of mental, behavioral, and neurodevelopmental disorders at baseline, which were treated as potential confounders. However, it should also be noted that controlling for internalizing symptoms may result in over-adjustment due to a possible causal pathway from *NR3C1* methylation to internalizing symptoms to substance use.

Conclusively, our findings indicate that *NR3C1* methylation levels can predict substance use outcomes in adolescence. However, additional longitudinal studies are warranted to confirm these findings and to investigate the role of genes versus environment in affecting *NR3C1* methylation.

## Supplementary information

Supplemental tables and figures

## Data Availability

The code of the statistical analysis is available under request to the corresponding author (http://elena.raffetti@ki.se).
