# Supplementary material for: DNA methylation of the glucocorticoid receptor gene predicts substance use in adolescence: longitudinal data from over 1000 young individuals
Source: Transl Psychiatry. 2021 Sep 15;11:477. doi: 10.1038/s41398-021-01601-6 (PMC8443651; doi:10.1038/s41398-021-01601-6)
Supplement: Supplementary file 1 — Supplemental tables and figures [file 41398_2021_1601_MOESM1_ESM.docx]

**Table S1.** Recent use, lifetime use and duration of substance use.

|  |  | **Total (n=1041)**  **n (%)** | **Males (n=476)**  **n (%)** | **Females (n=565)**  **n (%)** |
| --- | --- | --- | --- | --- |
| **Recent use** |  |  |  |  |
| Cigarettes |  | 96 (9.2) | 32 (6.7) | 64 (11.3) |
| Snus |  | 56 (5.4) | 44 (9.2) | 12 (2.1) |
| Tobacco |  | 122 (11.7) | 55 (11.5) | 67 (11.9) |
| Alcohol |  | 103 (9.9) | 34 (7.1) | 69 (12.2) |
| Cannabis |  | 14 (1.3) | 5 (1.0) | 9 (1.6) |
| Any Substance |  | 166 (16.0) | 64 (13.5) | 102 (18.0) |
|  |  |  |  |  |
| **Lifetime use** |  |  |  |  |
| Cigarettes |  | 193 (18.5) | 76 (16.0) | 117 (20.7) |
| Snus |  | 118 (11.3) | 75 (15.8) | 43 (7.6) |
| Tobacco |  | 225 (21.6) | 99 (20.8) | 126 (22.3) |
| Alcohol |  | 486 (46.7) | 200 (42.0) | 286 (50.6) |
| Cannabis |  | 35 (3.4) | 18 (3.8) | 17 (3.0) |
| Any Substance |  | 512 (49.2) | 217 (45.6) | 295 (52.2) |
|  |  |  |  |  |
| **Duration of use** | number of years of use |  |  |  |
| Cigarettes | No use | 945 (90.8) | 444 (93.3) | 501 (88.7) |
|  | 1 | 66 (6.3) | 23 (4.8) | 43 (7.6) |
|  | 2 | 24 (2.3) | 8 (1.7) | 16 (2.8) |
|  | 3 | 6 (0.6) | 1 (0.2) | 5 (0.9) |
| Snus | No use | 985 (94.6) | 432 (90.8) | 553 (97.9) |
|  | 1 | 37 (3.6) | 29 (6.1) | 8 (1.4) |
|  | 2 | 17 (1.6) | 14 (2.9) | 3 (0.5) |
|  | 3 | 2 (0.2) | 1 (0.2) | 1 (0.2) |
| Tobacco | No use | 919 (88.3) | 421 (88.4) | 498 (88.1) |
|  | 1 | 79 (7.6) | 36 (7.6) | 43 (7.6) |
|  | 2 | 35 (3.4) | 16 (3.4) | 19 (3.4) |
|  | 3 | 8 (0.8) | 3 (0.6) | 5 (0.9) |
| Alcohol | No use | 938 (90.1) | 442 (92.9) | 496 (87.8) |
|  | 1 | 79 (7.6) | 30 (6.3) | 49 (8.7) |
|  | 2 | 22 (2.1) | 4 (0.8) | 18 (3.2) |
|  | 3 | 2 (0.2) | 0 (0.0) | 2 (0.4) |
| Cannabis | No use | 1003 (96.3) | 456 (95.8) | 547 (96.8) |
|  | 1 | 30 (2.9) | 15 (3.1) | 15 (2.7) |
|  | 2 | 7 (0.7) | 5 (1.1) | 2 (0.4) |
|  | 3 | 1 (0.1) | 0 (0.0) | 1 (0.2) |
| Any Substance | No use | 866 (83.2) | 406 (85.3) | 460 (81.4) |
|  | 1 | 107 (10.3) | 42 (8.8) | 65 (11.5) |
|  | 2 | 58 (5.6) | 25 (5.3) | 33 (5.8) |
|  | 3 | 10 (1.0) | 3 (0.6) | 7 (1.2) |

**Table S2.** *NR3C1* methylation at baseline for those without or with initiation of current use over three-year follow-up among adolescent participants in the KUPOL study, Sweden.

| **CpG site** | non-cigarette users (n= 945) % | cigarette users (n=96) % |  | non-snus users (n=985) % | snus users  (n=56) % |  | non-tobacco users  (n= 919) % | tobacco users  (n= 122) % |  | non-alcohol users  (n= 938) % | alcohol users  (n= 103) % |  | non-cannabis users  (n= 1027) % | cannabis users  (n= 14) % |  | non-substance users  (n= 875)  % | substance users  (n= 166)  % |
| --- | --- | --- | --- | --- | --- | --- | --- | --- | --- | --- | --- | --- | --- | --- | --- | --- | --- |
| **CpG1 unmethylated** | 82.4 | 83.3 |  | 82.4 | 83.3 |  | 82.4 | 83.6 |  | 82.5 | 82.5 |  | 82.5 | 85.7 |  | 82.4 | 83.1 |
| **CpG1 low** | 9.3 | 6.3 |  | 9.0 | 8.9 |  | 9.4 | 6.6 |  | 9.5 | 4.9 |  | 9.2 | 0 |  | 9.5 | 6.6 |
| **CpG1 high** | 8.2 | 10.4 |  | 8.5 | 7.1 |  | 8.3 | 9.8 |  | 8.0 | 12.6 |  | 8.4 | 14.3 |  | 8.1 | 10.2 |
|  |  |  |  |  |  |  |  |  |  |  |  |  |  |  |  |  |  |
| **CpG2 unmethylated** | 88.1 | 81.3 |  | 87.3 | 91.1 |  | 87.9 | 84.4 |  | 87.8 | 84.5 |  | 87.7 | 71.4 |  | 88.0 | 85.1 |
| **CpG2 low** | 6.1 | 8.3 |  | 6.5 | 3.6 |  | 6.3 | 6.6 |  | 6.3 | 6.8 |  | 6.1 | 21.4 |  | 6.3 | 6.6 |
| **CpG2 high** | 5.7 | 10.4 |  | 6.2 | 5.4 |  | 5.8 | 9.0 |  | 5.8 | 8.7 |  | 6.1 | 7.1 |  | 5.7 | 8.4 |
|  |  |  |  |  |  |  |  |  |  |  |  |  |  |  |  |  |  |
| **CpG3 unmethylated** | 50.9 | 35.4 |  | 50.3 | 35.7 |  | 51.0 | 37.7 |  | 50.4 | 40.8 |  | 49.7 | 35.7 |  | 51.4 | 39.8 |
| **CpG3 low**  **CpG3 high** | 25.4 | 24.0 |  | 25.0 | 30.4 |  | 25.2 | 25.4 |  | 25.8 | 20.4 |  | 25.4 | 7.1 |  | 25.4 | 24.1 |
|  | 23.7 | 40.6 |  | 24.8 | 33.9 |  | 23.7 | 36.9 |  | 23.8 | 38.8 |  | 24.8 | 57.1 |  | 23.2 | 36.1 |
|  |  |  |  |  |  |  |  |  |  |  |  |  |  |  |  |  |  |
| **CpG4 unmethylated**  **CpG4 low** | 81.0 | 80.2 |  | 81.4 | 71.4 |  | 81.3 | 77.9 |  | 80.8 | 81.5 |  | 80.8 | 85.7 |  | 81.0 | 80.1 |
|  | 9.6 | 10.4 |  | 9.4 | 17.9 |  | 9.4 | 12.3 |  | 9.9 | 7.8 |  | 9.8 | 7.1 |  | 9.7 | 9.6 |
| **CpG4 high** | 9.4 | 9.4 |  | 9.3 | 10.7 |  | 9.4 | 9.8 |  | 9.3 | 10.7 |  | 9.4 | 7.1 |  | 9.3 | 10.2 |
|  |  |  |  |  |  |  |  |  |  |  |  |  |  |  |  |  |  |
| **CpG5 unmethylated** | 91.8 | 89.6 |  | 91.7 | 89.3 |  | 91.8 | 89.3 |  | 91.8 | 89.3 |  | 91.5 | 92.9 |  | 91.9 | 89.8 |
| **CpG5 low** | 4.2 | 2.1 |  | 3.9 | 7.1 |  | 4.0 | 4.1 |  | 4.2 | 2.9 |  | 4.1 | 0 |  | 4.0 | 4.2 |
| **CpG5 high** | 4.0 | 8.3 |  | 4.5 | 3.6 |  | 4.2 | 6.6 |  | 4.0 | 7.8 |  | 4.4 | 7.1 |  | 4.1 | 6.0 |
|  |  |  |  |  |  |  |  |  |  |  |  |  |  |  |  |  |  |
| **CpGs 1-5 unmethylated** | 39.8 | 29.2 |  | 39.4 | 28.6 |  | 39.8 | 31.1 |  | 39.0 | 36.9 |  | 38.9 | 35.7 |  | 39.8 | 33.7 |
| **CpGs 1-5 low** | 11.0 | 10.4 |  | 10.9 | 12.5 |  | 11.1 | 9.8 |  | 11.3 | 7.8 |  | 11.0 | 0 |  | 11.1 | 9.6 |
| **CpGs 1-5 high** | 49.2 | 60.4 |  | 49.8 | 58.9 |  | 49.1 | 59.0 |  | 49.7 | 55.3 |  | 50.1 | 64.3 |  | 49.1 | 56.6 |

Low methylation group: methylation levels below the median of detectable methylation and high methylation group: methylation levels above the median.

**Table S3.** Bi-variate association between *NR3C1* methylation at baseline and self-reports of recent use assessed over three years in adolescent participants of the KUPOL study, Sweden.

|  | **Cigarette use** |  | **Snus use** |  | **Tobacco use** |  | **Alcohol use** |  | **Cannabis use** |  | **Any Substance use** |
| --- | --- | --- | --- | --- | --- | --- | --- | --- | --- | --- | --- |
|  | **RR (95% CI)** |  | **RR (95% CI)** |  | **RR (95% CI)** |  | **RR (95% CI)** |  | **RR (95% CI)** |  | **RR (95% CI)** |
| **CpG1 low** | 0.68 (0.30, 1.55) |  | 0.97 (0.39,2.44) |  | 0.71 (0.34,1.45) |  | 0.53 (0.22,1.31) |  | - |  | 0.72 (0.39,1.32) |
| **CpG1 high** | 1.24 (0.64,2.40) |  | 0.83 (0.30,2.30) |  | 1.16 (0.64,2.12) |  | 1.49 (0.83,2.67) |  | 1.62 (0.36,7.23) |  | 1.22 (0.74,2.02) |
|  |  |  |  |  |  |  |  |  |  |  |  |
| **CpG2 low** | 1.41 (0.68,2.92) |  | 0.54 (0.13,2.20) |  | 1.06 (0.52,2.18) |  | 1.11 (0.51,2.39) |  | 4.17 (1.15,15.14) |  | 1.07 (0.58,1.97) |
| **CpG2 high** | 1.89 (0.98,3.65) |  | 0.84 (0.26,2.68) |  | 1.57 (0.84,2.92) |  | 1.47 (0.74,2.92) |  | 1.42 (0.18,11.08) |  | 1.46 (0.84,2.53) |
|  |  |  |  |  |  |  |  |  |  |  |  |
| **CpG3 low** | 1.34 (0.79,2.27) |  | 1.68 (0.88,3.21) |  | 1.34 (0.85,2.11) |  | 0.98 (0.58,1.66) |  | 0.39 (0.05,3.36) |  | 1.21 (0.81,1.79) |
| **CpG3 high** | 2.30 (1.45,3.65) |  | 1.87 (1.00,3.51) |  | 1.97 (1.30,2.96) |  | 1.89 (1.22,2.91) |  | 3.13 (1.03,9.58) |  | 1.84 (1.29,2.61) |
|  |  |  |  |  |  |  |  |  |  |  |  |
| **CpG4 low** | 1.08 (0.56,2.08) |  | 2.13 (1.06,4.25) |  | 1.33 (0.77,2.29) |  | 0.79 (0.38,1.64) |  | 0.69 (0.09,5.34) |  | 1.01 (0.60,1.70) |
| **CpG4 high** | 1.02 (0.51,2.03) |  | 1.29 (0.55,3.04) |  | 1.10 (0.60,2.01) |  | 1.12 (0.60,2.11) |  | 0.71 (0.09,5.48) |  | 1.12 (0.67,1.85) |
|  |  |  |  |  |  |  |  |  |  |  |  |
| **CpG5 low** | 0.53 (0.13,2.16) |  | 1.85 (0.67,5.12) |  | 1.07 (0.44,2.62) |  | 0.75 (0.24,2.37) |  | - |  | 1.10 (0.52,2.35) |
| **CpG5 high** | 2.01 (0.98,4.15) |  | 0.83 (0.20,3.41) |  | 1.58 (0.77,3.23) |  | 1.80 (0.87,3.70) |  | 1.59 (0.21,12.13) |  | 1.44 (0.76,2.73) |
|  |  |  |  |  |  |  |  |  |  |  |  |
| **CpGs 1-5 low** | 1.27 (0.62,2.61) |  | 1.55 (0.64,3.77) |  | 1.12 (0.58,2.14) |  | 0.74 (0.35,1.59) |  | - |  | 1.02 (0.58,1.77) |
| **CpGs 1-5 high** | 1.62 (1.03,2.54) |  | 1.61 (0.88,2.92) |  | 1.49 (1.00,2.20) |  | 1.16 (0.77,1.75) |  | 1.39 (0.47,4.14) |  | 1.32 (0.95,1.83) |

**Abbreviations** RR rate ratio, CI confidence interval. Methylation level 0% was considered as the reference category. Low methylation group: methylation levels below the median of detectable methylation and high methylation group: methylation levels above the median.

**Table S4.** E-values and lower confidence interval for the association between methylation levels at CpG3 and CpG1-5 sites and substance use for recent, lifetime and duration of use.

|  |  | **Cigarette use** |  | **Snus use** |  | **Tobacco use** |  | **Alcohol use** |  | **Cannabis use** |  | **Any Substance use** |
| --- | --- | --- | --- | --- | --- | --- | --- | --- | --- | --- | --- | --- |
|  |  | **E-value (lower 95% CI)** |  | **E-value (lower 95% CI)** |  | **E-value (lower 95% CI)** |  | **E-value (lower 95% CI)** |  | **E-value (lower 95% CI)** |  | **E-value (lower 95% CI)** |
| **Recent use** | **CpG3 high** | 4.03 (2.26) |  | 3.14 (1.00) |  | 3.35 (1.92) |  | 3.19 (1.74) |  | 5.71 (1.21) |  | 3.08 (1.90) |
|  | **CpGs 1-5 high** | 2.62 (1.21) |  | 2.61 (1.00) |  | 2.34 (1.00) |  | 1.59 (1.00) |  | 2.13 (1.00) |  | 1.97 (1.00) |
|  |  |  |  |  |  |  |  |  |  |  |  |  |
|  |  |  |  |  |  |  |  |  |  |  |  |  |
| **Lifetime use** | **CpG3 high** | 2.45 (1.46) |  | 2.41 (1.00) |  | 2.21 (1.31) |  | 1.51 (1.00) |  | 3.31 (1.00) |  | 1.49 (1.00) |
|  | **CpGs 1-5 high** | 1.74 (1.00) |  | 1.81 (1.00) |  | 1.67 (1.00) |  | 1.21 (1.00) |  | 1.24 (1.00) |  | 1.28 (1.00) |
|  |  |  |  |  |  |  |  |  |  |  |  |  |
|  |  |  |  |  |  |  |  |  |  |  |  |  |
| **Duration of use** | **CpG3 high** | 3.74 (2.28) |  | 3.19 (1.43) |  | 3.33 (2.10) |  | 2.87 (1.62) |  | 5.71 (1.16) |  | 2.81 (1.86) |
|  | **CpGs 1-5 high** | 2.43 (1.24) |  | 2.69 (1.00) |  | 1.74 (1.00) |  | 1.51 (1.00) |  | 2.13 (1.00) |  | - 1. 1.00) |

E-value represents the minimal strength of association on the RR scale that an unmeasured confounder would need to have, with both the predictor and the outcome, to fully explain the association between the two (Vanderweele TJ, Ding P. Sensitivity Analysis in Observational Research: Introducing the E-Value. Annals of internal medicine. 2017;167(4):268.)

**Table S5.** Association between *NR3C1* methylation at baseline and recent substance use assessed over three years in adolescent participants following adjustment for internalizing symptoms and parental history of mental, behavioral and neurodevelopmental disorder.

|  | **Cigarette use** |  | **Snus use** |  | **Tobacco use** |  | **Alcohol use** |  | **Cannabis use** |  | **Any Substance use** |
| --- | --- | --- | --- | --- | --- | --- | --- | --- | --- | --- | --- |
|  | **RR (95% CI)** |  | **RR (95% CI)** |  | **RR (95% CI)** |  | **RR (95% CI)** |  | **RR (95% CI)** |  | **RR (95% CI)** |
| **CpG1 low** | 0.75 (0.30,1.87) |  | 1.30 (0.51,3.31) |  | 0.81 (0.38,1.76) |  | 0.67 (0.27,1.67) |  | - |  | 0.86 (0.45,1.64) |
| **CpG1 high** | 1.16 (0.56,2.43) |  | 0.80 (0.25,2.62) |  | 1.16 (0.60,2.24) |  | 1.30 (0.67,2.52) |  | 0.75 (0.09,6.07) |  | 1.21 (0.69,2.10) |
|  |  |  |  |  |  |  |  |  |  |  |  |
| **CpG2 low** | 0.96 (0.39,2.40) |  | 0.32 (0.04,2.33) |  | 0.74 (0.30,1.83) |  | 1.02 (0.44,2.35) |  | 1.49 (0.19,11.94) |  | 0.75 (0.35,1.60) |
| **CpG2 high** | 1.77 (0.87,3.58) |  | 0.66 (0.16,2.75) |  | 1.54 (0.79,2.98) |  | 1.16 (0.53,2.53) |  | 1.09 (0.13,8.88) |  | 1.33 (0.73,2.42) |
|  |  |  |  |  |  |  |  |  |  |  |  |
| **CpG3 low** | 1.12 (0.62,2.03) |  | 1.41 (0.67,2.95) |  | 1.14 (0.68,1.91) |  | 0.81 (0.45,1.45) |  | - |  | 1.05 (0.67,1.63) |
| **CpG3 high** | 2.02 (1.22,3.35) |  | 1.87 (0.94,3.69) |  | 1.83 (1.17,2.87) |  | 1.69 (1.06,2.70) |  | 2.13 (0.64,7.04) |  | 1.70 (1.16,2.49) |
|  |  |  |  |  |  |  |  |  |  |  |  |
| **CpG4 low** | 0.88 (0.40,1.91) |  | 2.09 (0.96,4.52) |  | 1.28 (0.69,2.35) |  | 0.68 (0.30,1.57) |  | - |  | 0.98 (0.55,1.74) |
| **CpG4 high** | 0.84 (0.39,1.84) |  | 1.02 (0.36,2.89) |  | 1.02 (0.53,1.97) |  | 0.97 (0.48,1.95) |  | 0.66 (0.08,5.24) |  | 1.02 (0.58,1.78) |
|  |  |  |  |  |  |  |  |  |  |  |  |
| **CpG5 low** | 0.60 (0.15,2.47) |  | 2.25 (0.80,6.33) |  | 1.24 (0.50,3.05) |  | 0.83 (0.26,2.64) |  | - |  | 1.28 (0.60,2.75) |
| **CpG5 high** | 2.11 (0.97,4.60) |  | 0.50 (0.07,3.67) |  | 1.64 (0.76,3.54) |  | 1.48 (0.64,3.41) |  | 1.65 (0.21,13.08) |  | 1.35 (0.66,2.76) |
|  |  |  |  |  |  |  |  |  |  |  |  |
| **CpGs 1-5 low** | 1.11 (0.50,2.44) |  | 1.53 (0.59,3.99) |  | 1.05 (0.52,2.13) |  | 0.84 (0.39,1.81) |  | - |  | 1.03 (0.57,1.86) |
| **CpGs 1-5 high** | 1.30 (0.80,2.11) |  | 1.42 (0.74,2.73) |  | 1.29 (0.84,1.97) |  | 1.01 (0.65,1.58) |  | 0.84 (0.25,2.79) |  | 1.15 (0.80,1.66) |

**Abbreviations** RR rate ratio, CI confidence interval. Methylation level 0% was considered as the reference category. Low methylation group: methylation levels below the median of detectable methylation and high methylation group: methylation levels above the median.

**Table S6.** Association between *NR3C1* methylation at baseline and recent substance use assessed over three years in adolescent participants with Bonferroni corrected confidence intervals.

|  | **Cigarette use** |  | **Snus use** |  | **Tobacco use** |  | **Alcohol use** |  | **Cannabis use** |  | **Any Substance use** |
| --- | --- | --- | --- | --- | --- | --- | --- | --- | --- | --- | --- |
|  | **RR (95% CI)** |  | **RR (95% CI)** |  | **RR (95% CI)** |  | **RR (95% CI)** |  | **RR (95% CI)** |  | **RR (95% CI)** |
| **CpG1 low** | 0.68 (0.22,2.08) |  | 0.97 (0.28,3.38) |  | 0.71 (0.27,1.87) |  | 0.53 (0.16,1.80) |  | - |  | 0.72 (0.31,1.64) |
| **CpG1 high** | 1.24 (0.51,3.02) |  | 0.83 (0.21,3.29) |  | 1.16 (0.52,2.61) |  | 1.49 (0.68,3.28) |  | 1.62 (0.21,12.28) |  | 1.22 (0.62,2.41) |
|  |  |  |  |  |  |  |  |  |  |  |  |
| **CpG2 low** | 1.41 (0.53,3.77) |  | 0.53 (0.08,3.62) |  | 1.06 (0.40,2.80) |  | 1.11 (0.39,3.13) |  | 4.17 (0.73,23.88) |  | 1.07 (0.47,2.45) |
| **CpG2 high** | 1.89 (0.77,4.60) |  | 0.84 (0.17,4.04) |  | 1.57 (0.68,3.64) |  | 1.47 (0.58,3.71) |  | 1.42 (0.09,22.90) |  | 1.46 (0.69,3.07) |
|  |  |  |  |  |  |  |  |  |  |  |  |
| **CpG3 low** | 1.34 (0.66,2.74) |  | 1.69 (0.70,4.05) |  | 1.34 (0.72,2.48) |  | 0.98 (0.48,2.00) |  | 0.39 (0.02,7.17) |  | 1.21 (0.71,2.05) |
| **CpG3 high** | 2.30 (1.24,4.29) |  | 1.87 (0.80,4.38) |  | 1.97 (1.13,3.43) |  | 1.89 (1.05,3.39) |  | 3.13 (0.69,14.22) |  | 1.84 (1.14,2.95) |
|  |  |  |  |  |  |  |  |  |  |  |  |
| **CpG4 low** | 1.08 (0.44,2.62) |  | 2.12 (0.83,5.43) |  | 1.33 (0.64,2.77) |  | 0.79 (0.30,2.11) |  | 0.69 (0.04,10.97) |  | 1.01 (0.50,2.04) |
| **CpG4 high** | 1.02 (0.40,2.59) |  | 1.29 (0.40,4.11) |  | 1.10 (0.49,2.48) |  | 1.12 (0.48,2.63) |  | 0.71 (0.05,11.27) |  | 1.12 (0.56,2.21) |
|  |  |  |  |  |  |  |  |  |  |  |  |
| **CpG5 low** | 0.53 (0.08,3.54) |  | 1.85 (0.47,7.32) |  | 1.07 (0.32,3.59) |  | 0.75 (0.16,3.55) |  | - |  | 1.10 (0.39,3.07) |
| **CpG5 high** | 2.01 (0.75,5.36) |  | 0.83 (0.12,5.60) |  | 1.58 (0.60,4.16) |  | 1.79 (0.67,4.77) |  | 1.59 (0.10,24.88) |  | 1.44 (0.60,3.42) |
|  |  |  |  |  |  |  |  |  |  |  |  |
| **CpGs 1-5 low** | 1.28 (0.48,3.39) |  | 1.57 (0.47,5.21) |  | 1.13 (0.47,2.71) |  | 0.75 (0.27,2.11) |  | - |  | 1.02 (0.48,2.16) |
| **CpGs 1-5 high** | 1.62 (0.88,2.98) |  | 1.61 (0.72,3.60) |  | 1.49 (0.87,2.53) |  | 1.16 (0.67,2.02) |  | 1.39 (0.32,6.09) |  | 1.32 (0.84,2.06) |

**Abbreviations** RR rate ratio, CI confidence interval. Methylation level 0% was considered as the reference category. Low methylation group: methylation levels below the median of detectable methylation and high methylation group: methylation levels above the median.

|  | **Cigarette use** |  | **Snus use** |  | **Tobacco use** |  | **Alcohol use** |  | **Cannabis use** |  | **Any Substance use** |
| --- | --- | --- | --- | --- | --- | --- | --- | --- | --- | --- | --- |
|  | **RR (95% CI)** |  | **RR (95% CI)** |  | **RR (95% CI)** |  | **RR (95% CI)** |  | **RR (95% CI)** |  | **RR (95% CI)** |
| **CpG1 low** | 0.98 (0.30,3.22) |  | 1.20 (0.47,3.05) |  | 0.93 (0.37,2.35) |  | 0.29 (0.04,2.13) |  | - |  | 0.78 (0.31,1.94) |
| **CpG1 high** | 0.89 (0.21,3.75) |  | 0.63 (0.15,2.63) |  | 0.76 (0.24,2.43) |  | 1.20 (0.37,3.94) |  | - |  | 0.64 (0.20,2.04) |
|  |  |  |  |  |  |  |  |  |  |  |  |
| **CpG2 low** | 1.99 (0.69,5.69) |  | 0.60 (0.15,2.50) |  | 1.04 (0.37,2.88) |  | 1.30 (0.40,4.28) |  | 3.17 (0.35,28.39) |  | 0.87 (0.32,2.40) |
| **CpG2 high** | 1.24 (0.29,5.23) |  | 0.38 (0.05,2.76) |  | 0.65 (0.16,2.67) |  | 1.09 (0.26,4.58) |  | - |  | 0.54 (0.13,2.23) |
|  |  |  |  |  |  |  |  |  |  |  |  |
| **CpG3 low** | 3.09 (1.10,8.68) |  | 2.41 (1.15,5.06) |  | 2.22 (1.12,4.39) |  | 1.85 (0.75,4.55) |  | 2.04 (0.13,32.68) |  | 2.02 (1.08,3.78) |
| **CpG3 high** | 5.30 (2.09,13.44) |  | 2.25 (1.08,4.69) |  | 2.57 (1.35,4.90) |  | 2.75 (1.23,6.11) |  | 5.47 (0.57,52.62) |  | 2.35 (1.30,4.23) |
|  |  |  |  |  |  |  |  |  |  |  |  |
| **CpG4 low** | 1.85 (0.75,4.54) |  | 1.84 (0.85,3.98) |  | 2.06 (1.05,4.02) |  | 1.73 (0.71,4.22) |  | 1.79 (0.20,16.00) |  | 1.71 (0.89,3.29) |
| **CpG4 high** | 1.13 (0.34,3.78) |  | 1.06 (0.37,2.99) |  | 1.11 (0.44,2.82) |  | 1.04 (0.31,3.44) |  | - |  | 1.12 (0.48,2.62) |
|  |  |  |  |  |  |  |  |  |  |  |  |
| **CpG5 low** | 0.91 (0.12,6.68) |  | 2.01 (0.62,6.50) |  | 1.63 (0.51,5.23) |  | 0.86 (0.12,6.29) |  | - |  | 1.37 (0.43,4.38) |
| **CpG5 high** | 1.62 (0.39,6.80) |  | 0.56 (0.08,4.10) |  | 0.93 (0.23,3.82) |  | 1.48 (0.35,6.17) |  | - |  | 0.78 (0.19,3.20) |
|  |  |  |  |  |  |  |  |  |  |  |  |
| **CpGs 1-5 low** | 1.95 (0.49,7.81) |  | 1.78 (0.62,5.12) |  | 1.39 (0.50,3.86) |  | 0.77 (0.17,3.53) |  | - |  | 1.10 (0.41,2.98) |
| **CpGs 1-5 high** | 2.87 (1.17,7.04) |  | 1.90 (0.95,3.82) |  | 1.94 (1.05,3.60) |  | 1.63 (0.77,3.44) |  | 2.95 (0.33,26.38) |  | 1.73 (0.99,3.01) |

**Table S7.** Bi-variate association between *NR3C1* methylation at baseline and recent substance use assessed over three years in male participants of the KUPOL study, Sweden.

**Abbreviations** RR rate ratio, CI confidence interval. Methylation level 0% was considered as the reference category. Low methylation group: methylation levels below the median of detectable methylation and high methylation group: methylation levels above the median.

|  | **Cigarette use** |  | **Snus use** |  | **Tobacco use** |  | **Alcohol use** |  | **Cannabis use** |  | **Any Substance use** |
| --- | --- | --- | --- | --- | --- | --- | --- | --- | --- | --- | --- |
|  | **RR (95% CI)** |  | **RR (95% CI)** |  | **RR (95% CI)** |  | **RR (95% CI)** |  | **RR (95% CI)** |  | **RR (95% CI)** |
| **CpG1 low** | 0.52 (0.16,1.68) |  | - |  | 0.50 (0.16,1.61) |  | 0.68 (0.25,1.87) |  | - |  | 0.68 (0.29,1.55) |
| **CpG1 high** | 1.30 (0.62,2.73) |  | 1.70 (0.37,7.75) |  | 1.42 (0.70,2.87) |  | 1.52 (0.78,2.99) |  | 2.39 (0.50,11.49) |  | 1.47 (0.84,2.60) |
|  |  |  |  |  |  |  |  |  |  |  |  |
| **CpG2 low** | 1.12 (0.40,3.10) |  |  |  | 1.08 (0.39,2.97) |  | 1.03 (0.37,2.83) |  | 5.03 (1.02,24.92) |  | 1.25 (0.58,2.69) |
| **CpG2 high** | 2.11 (1.00,4.44) |  | 2.66 (0.58,12.13) |  | 2.31 (1.14,4.67) |  | 1.57 (0.72,3.45) |  | 2.16 (0.26,17.96) |  | 2.01 (1.10,3.68) |
|  |  |  |  |  |  |  |  |  |  |  |  |
| **CpG3 low** | 0.95 (0.50,1.81) |  | 0.54 (0.11,2.61) |  | 0.89 (0.47,1.67) |  | 0.71 (0.37,1.38) |  | - |  | 0.86 (0.52,1.45) |
| **CpG3 high** | 1.68 (0.96,2.94) |  | 0.90 (0.23,3.48) |  | 1.64 (0.95,2.82) |  | 1.66 (0.99,2.81) |  | 2.61 (0.70,9.71) |  | 1.64 (1.06,2.55) |
|  |  |  |  |  |  |  |  |  |  |  |  |
| **CpG4 low** | 0.71 (0.26,1.97) |  | 2.44 (0.52,11.47) |  | 0.69 (0.25,1.90) |  | 0.32 (0.08,1.32) |  | - |  | 0.56 (0.23,1.38) |
| **CpG4 high** | 0.97 (0.42,2.25) |  | 2.18 (0.46,10.25) |  | 1.09 (0.50,2.40) |  | 1.15 (0.55,2.42) |  | 1.07 (0.13,8.54) |  | 1.12 (0.60,2.09) |
|  |  |  |  |  |  |  |  |  |  |  |  |
| **CpG5 low** | 0.36 (0.05,2.60) |  | 2.07 (0.27,16.17) |  | 0.70 (0.17,2.88) |  | 0.68 (0.17,2.76) |  | - |  | 0.94 (0.34,2.55) |
| **CpG5 high** | 2.13 (0.92,4.94) |  | 1.94 (0.25,15.15) |  | 2.06 (0.89,4.76) |  | 1.87 (0.81,4.33) |  | 2.37 (0.30,18.94) |  | 1.79 (0.87,3.70) |
|  |  |  |  |  |  |  |  |  |  |  |  |
| **CpGs 1-5 low** | 1.05 (0.45,2.45) |  | 1.32 (0.26,6.80) |  | 0.96 (0.41,2.22) |  | 0.71 (0.29,1.71) |  | - |  | 0.95 (0.49,1.86) |
| **CpGs 1-5 high** | 1.29 (0.76,2.20) |  | 0.81 (0.23,2.79) |  | 1.22 (0.73,2.04) |  | 1.01 (0.61,1.65) |  | 1.00 (0.27,3.73) |  | 1.13 (0.74,1.71) |

**Table S8.** Bi-variate association between *NR3C1* methylation at baseline and recent substance use assessed over three years in female participants of the KUPOL study, Sweden.

**Abbreviations** RR rate ratio, CI confidence interval. Methylation level 0% was considered as the reference category. Low methylation group: methylation levels below the median of detectable methylation and high methylation group: methylation levels above the median.

**Table S9.** Bi-variate association between *NR3C1* methylation at baseline and lifetime substance use assessed over three years in adolescent participants of the KUPOL study, Sweden.

|  | **Cigarette use** |  | **Snus use** |  | **Tobacco use** |  | **Alcohol use** |  | **Cannabis use** |  | **Any Substance use** |
| --- | --- | --- | --- | --- | --- | --- | --- | --- | --- | --- | --- |
|  | **RR (95% CI)** |  | **RR (95% CI)** |  | **RR (95% CI)** |  | **RR (95% CI)** |  | **RR (95% CI)** |  | **RR (95% CI)** |
| **CpG1 low** | 0.52 (0.28,0.99) |  | 0.82 (0.42,1.63) |  | 0.59 (0.33,1.03) |  | 0.90 (0.65,1.24) |  | 0.31 (0.04,2.30) |  | 0.88 (0.64,1.21) |
| **CpG1 high** | 0.88 (0.52,1.48) |  | 0.87 (0.44,1.72) |  | 0.79 (0.48,1.32) |  | 1.10 (0.81,1.50) |  | 1.68 (0.65,4.35) |  | 1.09 (0.81,1.47) |
|  |  |  |  |  |  |  |  |  |  |  |  |
| **CpG2 low** | 1.09 (0.62,1.91) |  | 1.06 (0.51,2.17) |  | 0.98 (0.57,1.68) |  | 0.96 (0.67,1.39) |  | 1.47 (0.45,4.85) |  | 0.98 (0.69,1.40) |
| **CpG2 high** | 1.24 (0.72,2.13) |  | 0.96 (0.45,2.07) |  | 1.12 (0.66,1.89) |  | 1.01 (0.70,1.46) |  | 2.02 (0.71,5.75) |  | 1.06 (0.75,1.51) |
|  |  |  |  |  |  |  |  |  |  |  |  |
| **CpG3 low** | 1.09 (0.76,1.57) |  | 1.10 (0.69,1.74) |  | 1.02 (0.73,1.43) |  | 0.96 (0.77,1.20) |  | 0.65 (0.24,1.79) |  | 0.94 (0.75,1.16) |
| **CpG3 high** | 1.54 (1.11,2.14) |  | 1.52 (1.00,2.31) |  | 1.43 (1.06,1.94) |  | 1.13 (0.91,1.39) |  | 1.95 (0.95,3.99) |  | 1.12 (0.91,1.38) |
|  |  |  |  |  |  |  |  |  |  |  |  |
| **CpG4 low** | 1.06 (0.66,1.68) |  | 1.77 (1.06,2.93) |  | 1.11 (0.72,1.70) |  | 1.02 (0.76,1.38) |  | 0.84 (0.26,2.74) |  | 1.01 (0.75,1.35) |
| **CpG4 high** | 0.88 (0.53,1.47) |  | 1.30 (0.72,2.33) |  | 1.01 (0.65,1.59) |  | 1.08 (0.80,1.46) |  | 0.57 (0.14,2.38) |  | 1.14 (0.86,1.52) |
|  |  |  |  |  |  |  |  |  |  |  |  |
| **CpG5 low** | 1.05 (0.52,2.14) |  | 1.32 (0.58,3.00) |  | 1.15 (0.61,2.17) |  | 1.05 (0.67,1.64) |  | 0.73 (0.10,5.36) |  | 1.05 (0.68,1.62) |
| **CpG5 high** | 1.38 (0.75,2.53) |  | 1.39 (0.65,2.98) |  | 1.28 (0.71,2.29) |  | 1.21 (0.81,1.81) |  | 1.99 (0.61,6.51) |  | 1.17 (0.79,1.74) |
|  |  |  |  |  |  |  |  |  |  |  |  |
| **CpGs 1-5 low** | 0.86 (0.51,1.47) |  | 1.26 (0.68,2.31) |  | 0.96 (0.60,1.54) |  | 0.82 (0.60,1.13) |  | 0.50 (0.11,2.21) |  | 0.85 (0.63,1.16) |
| **CpGs 1-5 high** | 1.22 (0.90,1.65) |  | 1.25 (0.84,1.86) |  | 1.19 (0.90,1.58) |  | 1.03 (0.86,1.25) |  | 1.04 (0.52,2.08) |  | 1.05 (0.87,1.26) |

**Abbreviations** RR rate ratio, CI confidence interval. Methylation level 0% was considered as the reference category. Low methylation group: methylation levels below the median of detectable methylation and high methylation group: methylation levels above the median.

**Table S10.** Association between *NR3C1* methylation at baseline and lifetime substance use assessed over three years in adolescent participants following adjustment for internalizing symptoms and parental history of mental, behavioral and neurodevelopmental disorder.

|  | **Cigarette use** |  | **Snus use** |  | **Tobacco use** |  | **Alcohol use** |  | **Cannabis use** |  | **Any Substance use** |
| --- | --- | --- | --- | --- | --- | --- | --- | --- | --- | --- | --- |
|  | **RR (95% CI)** |  | **RR (95% CI)** |  | **RR (95% CI)** |  | **RR (95% CI)** |  | **RR (95% CI)** |  | **RR (95% CI)** |
| **CpG1 low** | 0.58 (0.28,1.18) |  | 0.96 (0.47,1.99) |  | 0.68 (0.37,1.26) |  | 0.93 (0.64,1.34) |  | 0.41 (0.06,3.02) |  | 0.94 (0.66,1.35) |
| **CpG1 high** | 0.88 (0.49,1.60) |  | 0.71 (0.31,1.64) |  | 0.83 (0.47,1.46) |  | 1.04 (0.74,1.48) |  | 1.46 (0.50,4.25) |  | 1.08 (0.77,1.51) |
|  |  |  |  |  |  |  |  |  |  |  |  |
| **CpG2 low** | 0.84 (0.43,1.66) |  | 0.76 (0.31,1.87) |  | 0.79 (0.42,1.51) |  | 0.87 (0.57,1.31) |  | 1.12 (0.26,4.77) |  | 0.90 (0.60,1.34) |
| **CpG2 high** | 1.19 (0.66,2.15) |  | 0.78 (0.32,1.93) |  | 1.12 (0.63,1.97) |  | 0.94 (0.62,1.42) |  | 1.92 (0.65,5.66) |  | 1.03 (0.70,1.53) |
|  |  |  |  |  |  |  |  |  |  |  |  |
| **CpG3 low** | 1.06 (0.70,1.59) |  | 1.07 (0.64,1.80) |  | 0.96 (0.66,1.40) |  | 0.95 (0.74,1.22) |  | 0.71 (0.23,2.23) |  | 0.92 (0.72,1.18) |
| **CpG3 high** | 1.47 (1.02,2.12) |  | 1.55 (0.98,2.47) |  | 1.36 (0.96,1.91) |  | 1.16 (0.92,1.47) |  | 2.35 (1.06,5.19) |  | 1.16 (0.92,1.46) |
|  |  |  |  |  |  |  |  |  |  |  |  |
| **CpG4 low** | 0.96 (0.56,1.65) |  | 1.79 (1.02,3.12) |  | 1.09 (0.68,1.76) |  | 1.05 (0.75,1.46) |  | 0.63 (0.15,2.67) |  | 1.02 (0.73,1.42) |
| **CpG4 high** | 0.73 (0.40,1.32) |  | 0.91 (0.44,1.88) |  | 0.87 (0.52,1.47) |  | 0.99 (0.71,1.39) |  | 0.57 (0.13,2.42) |  | 1.04 (0.75,1.44) |
|  |  |  |  |  |  |  |  |  |  |  |  |
| **CpG5 low** | 0.90 (0.40,2.03) |  | 1.29 (0.52,3.19) |  | 1.07 (0.53,2.19) |  | 1.03 (0.62,1.70) |  | 0.80 (0.11,5.90) |  | 1.05 (0.65,1.71) |
| **CpG5 high** | 1.39 (0.70,2.72) |  | 1.18 (0.48,2.91) |  | 1.33 (0.70,2.52) |  | 1.19 (0.76,1.87) |  | 2.14 (0.64,7.14) |  | 1.18 (0.76,1.83) |
|  |  |  |  |  |  |  |  |  |  |  |  |
| **CpGs 1-5 low** | 0.85 (0.48,1.53) |  | 1.17 (0.59,2.31) |  | 0.96 (0.57,1.61) |  | 0.87 (0.62,1.24) |  | 0.72 (0.16,3.27) |  | 0.89 (0.64,1.26) |
| **CpGs 1-5 high** | 1.07 (0.76,1.50) |  | 1.16 (0.75,1.80) |  | 1.08 (0.78,1.47) |  | 1.00 (0.81,1.24) |  | 1.26 (0.57,2.75) |  | 1.04 (0.84,1.28) |

**Abbreviations** RR rate ratio, CI confidence interval. Methylation level 0% was considered as the reference category. Low methylation group: methylation levels below the median of detectable methylation and high methylation group: methylation levels above the median.

**Table S11.** Association between *NR3C1* methylation at baseline and lifetime substance use assessed over three years in adolescent participants with Bonferroni corrected confidence intervals.

|  | **Cigarette use** |  | **Snus use** |  | **Tobacco use** |  | **Alcohol use** |  | **Cannabis use** |  | **Any Substance use** |
| --- | --- | --- | --- | --- | --- | --- | --- | --- | --- | --- | --- |
|  | **RR (95% CI)** |  | **RR (95% CI)** |  | **RR (95% CI)** |  | **RR (95% CI)** |  | **RR (95% CI)** |  | **RR (95% CI)** |
| **CpG1 low** | 0.52 (0.22,1.24) |  | 0.82 (0.33,2.07) |  | 0.59 (0.27,1.25) |  | 0.90 (0.58,1.39) |  | 0.31 (0.02,4.64) |  | 0.88 (0.58,1.36) |
| **CpG1 high** | 0.88 (0.43,1.79) |  | 0.87 (0.35,2.19) |  | 0.79 (0.40,1.58) |  | 1.10 (0.72,1.67) |  | 1.68 (0.47,6.08) |  | 1.09 (0.72,1.64) |
|  |  |  |  |  |  |  |  |  |  |  |  |
| **CpG2 low** | 1.09 (0.51,2.33) |  | 1.06 (0.40,2.80) |  | 0.98 (0.47,2.03) |  | 0.96 (0.58,1.59) |  | 1.47 (0.29,7.39) |  | 0.98 (0.60,1.59) |
| **CpG2 high** | 1.24 (0.59,2.59) |  | 0.96 (0.34,2.72) |  | 1.12 (0.55,2.27) |  | 1.01 (0.61,1.67) |  | 2.02 (0.49,8.33) |  | 1.06 (0.66,1.72) |
|  |  |  |  |  |  |  |  |  |  |  |  |
| **CpG3 low** | 1.09 (0.67,1.78) |  | 1.10 (0.59,2.04) |  | 1.02 (0.65,1.61) |  | 0.96 (0.71,1.30) |  | 0.65 (0.17,2.56) |  | 0.94 (0.70,1.25) |
| **CpG3 high** | 1.54 (0.99,2.40) |  | 1.52 (0.86,2.67) |  | 1.43 (0.95,2.16) |  | 1.13 (0.84,1.50) |  | 1.95 (0.74,5.14) |  | 1.12 (0.85,1.49) |
|  |  |  |  |  |  |  |  |  |  |  |  |
| **CpG4 low** | 1.06 (0.56,1.98) |  | 1.77 (0.89,3.51) |  | 1.11 (0.62,1.97) |  | 1.02 (0.68,1.53) |  | 0.84 (0.17,4.16) |  | 1.01 (0.68,1.50) |
| **CpG4 high** | 0.88 (0.44,1.76) |  | 1.30 (0.59,2.86) |  | 1.01 (0.55,1.87) |  | 1.08 (0.72,1.62) |  | 0.57 (0.08,3.94) |  | 1.14 (0.78,1.68) |
|  |  |  |  |  |  |  |  |  |  |  |  |
| **CpG5 low** | 1.05 (0.40,2.75) |  | 1.32 (0.43,4.01) |  | 1.15 (0.49,2.72) |  | 1.05 (0.57,1.93) |  | 0.73 (0.05,10.84) |  | 1.05 (0.58,1.89) |
| **CpG5 high** | 1.38 (0.60,3.14) |  | 1.39 (0.49,3.90) |  | 1.28 (0.58,2.81) |  | 1.21 (0.70,2.08) |  | 1.99 (0.40,9.89) |  | 1.17 (0.69,2.00) |
|  |  |  |  |  |  |  |  |  |  |  |  |
| **CpGs 1-5 low** | 0.86 (0.42,1.77) |  | 1.26 (0.55,2.87) |  | 0.96 (0.51,1.82) |  | 0.82 (0.53,1.26) |  | 0.50 (0.07,3.73) |  | 0.85 (0.56,1.29) |
| **CpGs 1-5 high** | 1.22 (0.81,1.84) |  | 1.25 (0.73,2.14) |  | 1.19 (0.82,1.74) |  | 1.03 (0.80,1.33) |  | 1.04 (0.41,2.65) |  | 1.05 (0.82,1.35) |

**Abbreviations** RR rate ratio, CI confidence interval. Methylation level 0% was considered as the reference category. Low methylation group: methylation levels below the median of detectable methylation and high methylation group: methylation levels above the median.

|  | **Cigarette use** |  | **Snus use** |  | **Tobacco use** |  | **Alcohol use** |  | **Cannabis use** |  | **Any Substance use** |
| --- | --- | --- | --- | --- | --- | --- | --- | --- | --- | --- | --- |
|  | **RR (95% CI)** |  | **RR (95% CI)** |  | **RR (95% CI)** |  | **RR (95% CI)** |  | **RR (95% CI)** |  | **RR (95% CI)** |
| **CpG1 low** | 0.64 (0.26,1.58) |  | 0.95 (0.43,2.06) |  | 0.69 (0.32,1.48) |  | 1.10 (0.69,1.75) |  | - |  | 1.03 (0.66,1.63) |
| **CpG1 high** | 0.70 (0.26,1.92) |  | 0.53 (0.17,1.70) |  | 0.66 (0.27,1.62) |  | 1.33 (0.82,2.17) |  | - |  | 1.26 (0.78,2.01) |
|  |  |  |  |  |  |  |  |  |  |  |  |
| **CpG2 low** | 0.73 (0.27,2.00) |  | 0.71 (0.26,1.94) |  | 0.54 (0.20,1.47) |  | 0.90 (0.51,1.58) |  | 1.69 (0.39,7.38) |  | 0.83 (0.47,1.46) |
| **CpG2 high** | 0.68 (0.21,2.16) |  | 0.44 (0.11,1.80) |  | 0.67 (0.25,1.82) |  | 0.70 (0.34,1.42) |  | 1.06 (0.14,8.02) |  | 0.79 (0.42,1.49) |
|  |  |  |  |  |  |  |  |  |  |  |  |
| **CpG3 low** | 1.62 (0.91,2.86) |  | 1.34 (0.75,2.37) |  | 1.32 (0.80,2.17) |  | 0.94 (0.66,1.34) |  | 0.50 (0.11,2.38) |  | 0.90 (0.64,1.26) |
| **CpG3 high** | 1.92 (1.13,3.26) |  | 1.58 (0.94,2.68) |  | 1.59 (1.00,2.51) |  | 1.09 (0.79,1.51) |  | 1.82 (0.68,4.84) |  | 1.14 (0.83,1.55) |
|  |  |  |  |  |  |  |  |  |  |  |  |
| **CpG4 low** | 1.62 (0.89,2.96) |  | 1.56 (0.83,2.90) |  | 1.46 (0.84,2.54) |  | 1.15 (0.75,1.75) |  | 0.90 (0.21,3.90) |  | 1.16 (0.77,1.74) |
| **CpG4 high** | 0.73 (0.29,1.83) |  | 0.90 (0.39,2.09) |  | 1.03 (0.52,2.06) |  | 1.04 (0.65,1.68) |  | - |  | 1.20 (0.78,1.86) |
|  |  |  |  |  |  |  |  |  |  |  |  |
| **CpG5 low** | 1.12 (0.35,3.56) |  | 1.16 (0.37,3.69) |  | 1.51 (0.62,3.72) |  | 0.97 (0.45,2.06) |  | - |  | 1.03 (0.51,2.08) |
| **CpG5 high** | 1.00 (0.31,3.16) |  | 0.96 (0.30,3.04) |  | 1.01 (0.37,2.75) |  | 1.01 (0.50,2.06) |  | - |  | 1.03 (0.53,2.01) |
|  |  |  |  |  |  |  |  |  |  |  |  |
| **CpGs 1-5 low** | 1.12 (0.48,2.61) |  | 1.38 (0.65,2.95) |  | 1.16 (0.57,2.34) |  | 0.76 (0.45,1.31) |  | - |  | 0.83 (0.50,1.36) |
| **CpGs 1-5 high** | 1.39 (0.85,2.28) |  | 1.14 (0.70,1.87) |  | 1.21 (0.79,1.85) |  | 1.06 (0.79,1.42) |  | 0.92 (0.36,2.32) |  | 1.05 (0.79,1.39) |

**Table S12.** Bi-variate association between *NR3C1* methylation at baseline and lifetime substance use assessed over three years in male participants of the KUPOL study, Sweden.

**Abbreviations** RR rate ratio, CI confidence interval. Methylation level 0% was considered as the reference category. Low methylation group: methylation levels below the median of detectable methylation and high methylation group: methylation levels above the median.

|  | **Cigarette use** |  | **Snus use** |  | **Tobacco use** |  | **Alcohol use** |  | **Cannabis use** |  | **Any Substance use** |
| --- | --- | --- | --- | --- | --- | --- | --- | --- | --- | --- | --- |
|  | **RR (95% CI)** |  | **RR (95% CI)** |  | **RR (95% CI)** |  | **RR (95% CI)** |  | **RR (95% CI)** |  | **RR (95% CI)** |
| **CpG1 low** | 0.45 (0.18,1.10) |  | 0.54 (0.13,2.25) |  | 0.50 (0.22,1.14) |  | 0.76 (0.48,1.19) |  | 0.85 (0.11,6.59) |  | 0.77 (0.50,1.21) |
| **CpG1 high** | 0.93 (0.50,1.74) |  | 1.46 (0.62,3.48) |  | 0.87 (0.47,1.62) |  | 0.95 (0.64,1.42) |  | 3.83 (1.33,11.04) |  | 0.98 (0.66,1.44) |
|  |  |  |  |  |  |  |  |  |  |  |  |
| **CpG2 low** | 1.41 (0.71,2.79) |  | 1.76 (0.63,4.97) |  | 1.43 (0.75,2.74) |  | 1.04 (0.63,1.69) |  | 1.14 (0.15,8.74) |  | 1.13 (0.71,1.80) |
| **CpG2 high** | 1.58 (0.85,2.95) |  | 1.98 (0.77,5.06) |  | 1.48 (0.79,2.75) |  | 1.20 (0.77,1.85) |  | 2.98 (0.85,10.46) |  | 1.25 (0.81,1.91) |
|  |  |  |  |  |  |  |  |  |  |  |  |
| **CpG3 low** | 0.85 (0.53,1.36) |  | 0.82 (0.38,1.79) |  | 0.84 (0.53,1.32) |  | 0.98 (0.73,1.30) |  | 0.81 (0.21,3.15) |  | 0.96 (0.73,1.27) |
| **CpG3 high** | 1.38 (0.90,2.09) |  | 1.32 (0.66,2.64) |  | 1.34 (0.89,2.01) |  | 1.17 (0.89,1.55) |  | 2.08 (0.73,5.92) |  | 1.13 (0.85,1.49) |
|  |  |  |  |  |  |  |  |  |  |  |  |
| **CpG4 low** | 0.67 (0.31,1.43) |  | 1.95 (0.81,4.68) |  | 0.81 (0.41,1.59) |  | 0.94 (0.61,1.44) |  | 0.69 (0.09,5.24) |  | 0.90 (0.58,1.37) |
| **CpG4 high** | 0.97 (0.52,1.81) |  | 2.05 (0.90,4.67) |  | 1.00 (0.55,1.82) |  | 1.11 (0.76,1.64) |  | 1.22 (0.28,5.35) |  | 1.11 (0.76,1.61) |
|  |  |  |  |  |  |  |  |  |  |  |  |
| **CpG5 low** | 0.99 (0.41,2.44) |  | 1.72 (0.53,5.60) |  | 0.92 (0.38,2.26) |  | 1.09 (0.62,1.90) |  | 1.59 (0.21,12.16) |  | 1.05 (0.60,1.84) |
| **CpG5 high** | 1.59 (0.78,3.27) |  | 2.21 (0.79,6.20) |  | 1.47 (0.72,3.02) |  | 1.31 (0.80,2.14) |  | 4.36 (1.24,15.30) |  | 1.25 (0.77,2.04) |
|  |  |  |  |  |  |  |  |  |  |  |  |
| **CpGs 1-5 low** | 0.72 (0.36,1.44) |  | 1.19 (0.43,3.30) |  | 0.84 (0.45,1.58) |  | 0.84 (0.57,1.25) |  | 1.10 (0.22,5.45) |  | 0.86 (0.58,1.27) |
| **CpGs 1-5 high** | 1.14 (0.78,1.68) |  | 1.40 (0.73,2.72) |  | 1.19 (0.82,1.72) |  | 1.03 (0.80,1.31) |  | 1.20 (0.43,3.37) |  | 1.06 (0.83,1.35) |

**Table S13.** Bi-variate association between *NR3C1* methylation at baseline and lifetime substance use assessed over three years in female participants of the KUPOL study, Sweden.

**Abbreviations** RR rate ratio, CI confidence interval. Methylation level 0% was considered as the reference category. Low methylation group: methylation levels below the median of detectable methylation and high methylation group: methylation levels above the median.

**Table S14.** Bi-variate association between *NR3C1* methylation levels at baseline and duration of substance use (per year increase) assessed over three years in adolescent participants of the KUPOL study, Sweden.

|  | **Cigarette use** |  | **Snus use** |  | **Tobacco use** |  | **Alcohol use** |  | **Cannabis use** |  | **Any Substance use** |
| --- | --- | --- | --- | --- | --- | --- | --- | --- | --- | --- | --- |
|  | **RR (95% CI)** |  | **RR (95% CI)** |  | **RR (95% CI)** |  | **RR (95% CI)** |  | **RR (95% CI)** |  | **RR (95% CI)** |
| **CpG1 low** | 0.58 (0.27,1.24) |  | 1.00 (0.46,2.18) |  | 0.70 (0.38,1.29) |  | 0.50 (0.22,1.14) |  | - |  | 0.69 (0.41,1.17) |
| **CpG1 high** | 1.23 (0.71,2.15) |  | 0.92 (0.40,2.11) |  | 1.22 (0.75,1.99) |  | 1.25 (0.72,2.19) |  | 1.62 (0.36,7.26) |  | 1.23 (0.81,1.87) |
|  |  |  |  |  |  |  |  |  |  |  |  |
| **CpG2 low** | 1.27 (0.66,2.42) |  | 0.39 (0.10,1.61) |  | 0.94 (0.49,1.78) |  | 0.87 (0.41,1.87) |  | 4.14 (1.14,15.03) |  | 0.95 (0.55,1.63) |
| **CpG2 high** | 1.70 (0.96,3.02) |  | 1.02 (0.41,2.52) |  | 1.55 (0.92,2.60) |  | 1.41 (0.76,2.62) |  | 1.42 (0.18,11.11) |  | 1.47 (0.94,2.31) |
|  |  |  |  |  |  |  |  |  |  |  |  |
| **CpG3 low** | 1.26 (0.81,1.99) |  | 1.74 (1.00,3.02) |  | 1.38 (0.94,2.02) |  | 1.07 (0.68,1.68) |  | 0.39 (0.05,3.36) |  | 1.24 (0.90,1.72) |
| **CpG3 high** | 2.16 (1.46,3.20) |  | 1.89 (1.10,3.23) |  | 1.96 (1.38,2.77) |  | 1.74 (1.17,2.57) |  | 3.13 (1.02,9.58) |  | 1.71 (1.27,2.30) |
|  |  |  |  |  |  |  |  |  |  |  |  |
| **CpG4 low** | 0.94 (0.52,1.71) |  | 2.16 (1.20,3.89) |  | 1.33 (0.84,2.10) |  | 0.78 (0.41,1.49) |  | 0.69 (0.09,5.34) |  | 1.06 (0.70,1.63) |
| **CpG4 high** | 1.13 (0.65,1.98) |  | 1.43 (0.71,2.90) |  | 1.30 (0.81,2.08) |  | 0.96 (0.53,1.75) |  | 0.72 (0.09,5.50) |  | 1.19 (0.79,1.79) |
|  |  |  |  |  |  |  |  |  |  |  |  |
| **CpG5 low** | 0.78 (0.29,2.10) |  | 2.00 (0.87,4.61) |  | 1.34 (0.69,2.63) |  | 0.79 (0.29,2.14) |  | - |  | 1.18 (0.64,2.17) |
| **CpG5 high** | 1.95 (1.05,3.61) |  | 0.91 (0.29,2.90) |  | 1.64 (0.91,2.94) |  | 1.80 (0.94,3.44) |  | 1.59 (0.21,12.17) |  | 1.57 (0.94,2.61) |
|  |  |  |  |  |  |  |  |  |  |  |  |
| **CpGs 1-5 low** | 1.15 (0.62,2.15) |  | 1.29 (0.57,2.89) |  | 1.00 (0.57,1.78) |  | 0.81 (0.42,1.56) |  | - |  | 0.93 (0.57,1.50) |
| **CpGs 1-5 high** | 1.53 (1.04,2.23) |  | 1.65 (0.99,2.74) |  | 1.22 (0.73,2.04) |  | 1.13 (0.78,1.63) |  | 1.39 (0.47,4.15) |  | 1.30 (0.99,1.71) |

**Abbreviations** RR risk ratio, CI confidence interval. Methylation level 0% was considered as the reference category. Low methylation group: methylation levels below the median of detectable methylation and high methylation group: methylation levels above the median

**Table S15.** Bi-variate association between *NR3C1* methylation levels at baseline and duration of substance use (per year increase) assessed over three years in adolescent participants following adjustment for internalizing symptoms and parental history of mental, behavioral and neurodevelopmental disorders.

|  | **Cigarette use** |  | **Snus use** |  | **Tobacco use** |  | **Alcohol use** |  | **Cannabis use** |  | **Any Substance use** |
| --- | --- | --- | --- | --- | --- | --- | --- | --- | --- | --- | --- |
|  | **RR (95% CI)** |  | **RR (95% CI)** |  | **RR (95% CI)** |  | **RR (95% CI)** |  | **RR (95% CI)** |  | **RR (95% CI)** |
| **CpG1 low** | 0.67 (0.29,1.52) |  | 1.29 (0.58,2.83) |  | 0.84 (0.44,1.59) |  | 0.64 (0.28,1.46) |  | - |  | 0.84 (0.49,1.46) |
| **CpG1 high** | 1.14 (0.62,2.10) |  | 0.93 (0.37,2.35) |  | 1.22 (0.72,2.07) |  | 1.10 (0.58,2.05) |  | 0.76 (0.10,6.15) |  | 1.22 (0.78,1.92) |
|  |  |  |  |  |  |  |  |  |  |  |  |
| **CpG2 low** | 0.69 (0.28,1.71) |  | 0.23 (0.03,1.66) |  | 0.53 (0.22,1.30) |  | 0.81 (0.35,1.85) |  | 1.50 (0.19,11.98) |  | 0.61 (0.30,1.24) |
| **CpG2 high** | 1.47 (0.80,2.71) |  | 0.91 (0.33,2.53) |  | 1.45 (0.84,2.50) |  | 1.17 (0.59,2.33) |  | 1.10 (0.14,8.99) |  | 1.34 (0.83,2.16) |
|  |  |  |  |  |  |  |  |  |  |  |  |
| **CpG3 low** | 1.15 (0.69,1.90) |  | 1.55 (0.84,2.87) |  | 1.27 (0.83,1.95) |  | 0.91 (0.55,1.51) |  | - |  | 1.10 (0.77,1.59) |
| **CpG3 high** | 1.95 (1.27,2.99) |  | 1.94 (1.09,3.47) |  | 1.88 (1.29,2.75) |  | 1.63 (1.07,2.48) |  | 2.15 (0.65,7.11) |  | 1.61 (1.17,2.22) |
|  |  |  |  |  |  |  |  |  |  |  |  |
| **CpG4 low** | 0.72 (0.35,1.48) |  | 2.24 (1.19,4.24) |  | 1.26 (0.76,2.11) |  | 0.63 (0.29,1.36) |  | - |  | 0.98 (0.61,1.59) |
| **CpG4 high** | 0.82 (0.42,1.57) |  | 1.29 (0.58,2.86) |  | 1.11 (0.66,1.88) |  | 0.84 (0.44,1.62) |  | 0.67 (0.08,5.31) |  | 1.02 (0.65,1.61) |
|  |  |  |  |  |  |  |  |  |  |  |  |
| **CpG5 low** | 0.85 (0.31,2.33) |  | 2.32 (1.00,5.41) |  | 1.50 (0.76,2.95) |  | 0.87 (0.32,2.36) |  | - |  | 1.31 (0.71,2.41) |
| **CpG5 high** | 1.96 (1.02,3.77) |  | 0.70 (0.17,2.87) |  | 1.68 (0.90,3.11) |  | 1.59 (0.77,3.28) |  | 1.67 (0.21,13.23) |  | 1.52 (0.88,2.63) |
|  |  |  |  |  |  |  |  |  |  |  |  |
| **CpGs 1-5 low** | 1.11 (0.56,2.18) |  | 1.31 (0.55,3.12) |  | 1.01 (0.55,1.87) |  | 0.94 (0.48,1.82) |  | - |  | 0.96 (0.58,1.60) |
| **CpGs 1-5 high** | 1.26 (0.84,1.91) |  | 1.57 (0.90,2.72) |  | 1.39 (0.97,1.99) |  | 1.02 (0.68,1.52) |  | 0.86 (0.26,2.84) |  | 1.16 (0.86,1.56) |

**Abbreviations** RR risk ratio, CI confidence interval. Methylation level 0% was considered as the reference category. Low methylation group: methylation levels below the median of detectable methylation and high methylation group: methylation levels above the median.

**Table S16.** Bi-variate association between *NR3C1* methylation levels at baseline and duration of substance use (per year increase) assessed over three years in adolescent participants with Bonferroni corrected confidence intervals.

|  | **Cigarette use** |  | **Snus use** |  | **Tobacco use** |  | **Alcohol use** |  | **Cannabis use** |  | **Any Substance use** |
| --- | --- | --- | --- | --- | --- | --- | --- | --- | --- | --- | --- |
|  | **RR (95% CI)** |  | **RR (95% CI)** |  | **RR (95% CI)** |  | **RR (95% CI)** |  | **RR (95% CI)** |  | **RR (95% CI)** |
| **CpG1 low** | 0.58 (0.20,1.62) |  | 1.00 (0.35,2.87) |  | 0.70 (0.30,1.60) |  | 0.50 (0.17,1.53) |  | - |  | 0.69 (0.34,1.41) |
| **CpG1 high** | 1.23 (0.58,2.61) |  | 0.91 (0.29,2.84) |  | 1.22 (0.63,2.37) |  | 1.25 (0.59,2.66) |  | 1.62 (0.21,12.32) |  | 1.23 (0.70,2.16) |
|  |  |  |  |  |  |  |  |  |  |  |  |
| **CpG2 low** | 1.26 (0.53,3.04) |  | 0.39 (0.06,2.64) |  | 0.94 (0.39,2.23) |  | 0.87 (0.31,2.44) |  | 4.14 (0.72,23.70) |  | 0.95 (0.46,1.98) |
| **CpG2 high** | 1.70 (0.78,3.69) |  | 1.02 (0.30,3.47) |  | 1.55 (0.77,3.11) |  | 1.41 (0.61,3.26) |  | 1.42 (0.09,22.95) |  | 1.47 (0.80,2.70) |
|  |  |  |  |  |  |  |  |  |  |  |  |
| **CpG3 low** | 1.27 (0.69,2.34) |  | 1.75 (0.83,3.68) |  | 1.38 (0.83,2.32) |  | 1.08 (0.58,1.98) |  | 0.39 (0.02,7.18) |  | 1.24 (0.80,1.92) |
| **CpG3 high** | 2.16 (1.27,3.67) |  | 1.89 (0.91,3.91) |  | 1.96 (1.23,3.13) |  | 1.74 (1.02,2.95) |  | 3.13 (0.69,14.21) |  | 1.71 (1.15,2.55) |
|  |  |  |  |  |  |  |  |  |  |  |  |
| **CpG4 low** | 0.94 (0.42,2.11) |  | 2.16 (0.97,4.78) |  | 1.32 (0.71,2.47) |  | 0.78 (0.32,1.87) |  | 0.69 (0.04,10.97) |  | 1.06 (0.60,1.89) |
| **CpG4 high** | 1.13 (0.53,2.41) |  | 1.43 (0.55,3.72) |  | 1.30 (0.69,2.46) |  | 0.96 (0.43,2.16) |  | 0.72 (0.05,11.30) |  | 1.19 (0.68,2.07) |
|  |  |  |  |  |  |  |  |  |  |  |  |
| **CpG5 low** | 0.77 (0.20,2.98) |  | 2.00 (0.65,6.19) |  | 1.34 (0.54,3.33) |  | 0.79 (0.20,3.04) |  | - |  | 1.18 (0.52,2.68) |
| **CpG5 high** | 1.95 (0.84,4.49) |  | 0.91 (0.19,4.36) |  | 1.63 (0.74,3.62) |  | 1.80 (0.75,4.31) |  | 1.59 (0.10,24.95) |  | 1.57 (0.79,3.12) |
|  |  |  |  |  |  |  |  |  |  |  |  |
| **CpGs 1-5 low** | 1.16 (0.50,2.71) |  | 1.30 (0.43,3.89) |  | 1.01 (0.47,2.20) |  | 0.82 (0.34,1.99) |  | - |  | 0.93 (0.48,1.77) |
| **CpGs 1-5 high** | 1.53 (0.91,2.55) |  | 1.65 (0.83,3.27) |  | 1.53 (0.98,2.39) |  | 1.13 (0.69,1.85) |  | 1.39 (0.32,6.10) |  | 1.30 (0.89,1.88) |

**Abbreviations** RR risk ratio, CI confidence interval. Methylation level 0% was considered as the reference category. Low methylation group: methylation levels below the median of detectable methylation and high methylation group: methylation levels above the median.

|  | **Cigarette use** |  | **Snus use** |  | **Tobacco use** |  | **Alcohol use** |  | **Cannabis use** |  | **Any Substance use** |
| --- | --- | --- | --- | --- | --- | --- | --- | --- | --- | --- | --- |
|  | **RR (95% CI)** |  | **RR (95% CI)** |  | **RR (95% CI)** |  | **RR (95% CI)** |  | **RR (95% CI)** |  | **RR (95% CI)** |
| **CpG1 low** | 0.98 (0.35,2.76) |  | 1.21 (0.55,2.67) |  | 1.07 (0.51,2.23) |  | 0.26 (0.04,1.90) |  | - |  | 0.89 (0.43,1.85) |
| **CpG1 high** | 0.67 (0.16,2.78) |  | 0.47 (0.12,1.94) |  | 0.55 (0.17,1.74) |  | 1.06 (0.33,3.46) |  | - |  | 0.76 (0.31,1.88) |
|  |  |  |  |  |  |  |  |  |  |  |  |
| **CpG2 low** | 2.23 (0.94,5.31) |  | 0.44 (0.11,1.82) |  | 1.10 (0.48,2.53) |  | 1.15 (0.35,3.75) |  | 3.15 (0.35,28.20) |  | 1.08 (0.50,2.33) |
| **CpG2 high** | 0.94 (0.23,3.93) |  | 0.28 (0.04,2.03) |  | 0.46 (0.11,1.90) |  | 0.97 (0.23,4.05) |  | - |  | 0.59 (0.18,1.85) |
|  |  |  |  |  |  |  |  |  |  |  |  |
| **CpG3 low** | 2.48 (1.03,5.98) |  | 2.35 (1.27,4.33) |  | 2.30 (1.30,4.08) |  | 2.03 (0.88,4.67) |  | 2.04 (0.13,32.68) |  | 2.20 (1.30,3.71) |
| **CpG3 high** | 4.45 (2.05,9.66) |  | 1.82 (0.96,3.44) |  | 2.48 (1.43,4.30) |  | 2.65 (1.23,5.71) |  | 5.46 (0.57,52.50) |  | 2.43 (1.47,4.00) |
|  |  |  |  |  |  |  |  |  |  |  |  |
| **CpG4 low** | 1.38 (0.58,3.32) |  | 2.00 (1.05,3.78) |  | 1.99 (1.12,3.52) |  | 1.79 (0.78,4.09) |  | 1.78 (0.20,15.95) |  | 1.73 (1.00,2.99) |
| **CpG4 high** | 1.39 (0.54,3.57) |  | 1.00 (0.40,2.53) |  | 1.28 (0.61,2.68) |  | 0.92 (0.28,3.04) |  | - |  | 1.30 (0.67,2.53) |
|  |  |  |  |  |  |  |  |  |  |  |  |
| **CpG5 low** | 1.36 (0.33,5.65) |  | 1.88 (0.68,5.19) |  | 1.85 (0.75,4.58) |  | 1.52 (0.37,6.34) |  | - |  | 1.54 (0.62,3.79) |
| **CpG5 high** | 1.22 (0.29,5.05) |  | 0.42 (0.06,3.04) |  | 0.66 (0.16,2.70) |  | 1.36 (0.33,5.67) |  | - |  | 0.83 (0.26,2.61) |
|  |  |  |  |  |  |  |  |  |  |  |  |
| **CpGs 1-5 low** | 1.72 (0.53,5.59) |  | 1.45 (0.57,3.71) |  | 1.22 (0.49,3.06) |  | 0.70 (0.16,3.18) |  | - |  | 0.99 (0.40,2.42) |
| **CpGs 1-5 high** | 2.37 (1.12,5.02) |  | 1.75 (0.98,3.14) |  | 2.02 (1.19,3.41) |  | 1.67 (0.82,3.40) |  | 2.95 (0.33,26.37) |  | 1.90 (1.19,3.05) |

**Table S17.** Bi-variate association between *NR3C1* methylation levels at baseline and duration of substance use (per year increase) assessed over three years in male participants of the KUPOL study, Sweden.

**Abbreviations** RR risk ratio, CI confidence interval. Methylation level 0% was considered as the reference category. Low methylation group: methylation levels below the median of detectable methylation and high methylation group: methylation levels above the median.

|  | **Cigarette use** |  | **Snus use** |  | **Tobacco use** |  | **Alcohol use** |  | **Cannabis use** |  | **Any Substance use** |
| --- | --- | --- | --- | --- | --- | --- | --- | --- | --- | --- | --- |
|  | **RR (95% CI)** |  | **RR (95% CI)** |  | **RR (95% CI)** |  | **RR (95% CI)** |  | **RR (95% CI)** |  | **RR (95% CI)** |
| **CpG1 low** | 0.38 (0.12,1.19) |  | - |  | 0.36 (0.11,1.15) |  | 0.63 (0.25,1.55) |  | - |  | 0.55 (0.26,1.19) |
| **CpG1 high** | 1.34 (0.73,2.47) |  | 2.58 (0.84,7.91) |  | 1.61 (0.93,2.80) |  | 1.23 (0.65,2.31) |  | 2.39 (0.50,11.53) |  | 1.41 (0.88,2.26) |
|  |  |  |  |  |  |  |  |  |  |  |  |
| **CpG2 low** | 0.80 (0.29,2.18) |  | - |  | 0.77 (0.28,2.10) |  | 0.77 (0.28,2.10) |  | 4.99 (1.01,24.72) |  | 0.87 (0.40,1.86) |
| **CpG2 high** | 1.91 (1.01,3.59) |  | 4.00 (1.30,12.27) |  | 2.33 (1.32,4.12) |  | 1.50 (0.75,2.99) |  | 2.17 (0.26,18.00) |  | 1.93 (1.18,3.17) |
|  |  |  |  |  |  |  |  |  |  |  |  |
| **CpG3 low** | 0.98 (0.57,1.68) |  | 0.48 (0.10,2.24) |  | 0.91 (0.53,1.55) |  | 0.82 (0.47,1.42) |  | - |  | 0.87 (0.57,1.33) |
| **CpG3 high** | 1.66 (1.04,2.66) |  | 1.83 (0.66,5.04) |  | 1.69 (1.08,2.66) |  | 1.54 (0.97,2.45) |  | 2.61 (0.70,9.72) |  | 1.44 (1.00,2.09) |
|  |  |  |  |  |  |  |  |  |  |  |  |
| **CpG4 low** | 0.77 (0.34,1.77) |  | 1.75 (0.39,7.91) |  | 0.74 (0.32,1.70) |  | 0.37 (0.12,1.16) |  | - |  | 0.63 (0.31,1.29) |
| **CpG4 high** | 1.03 (0.52,2.05) |  | 3.12 (0.99,9.79) |  | 1.32 (0.72,2.42) |  | 0.98 (0.49,1.95) |  | 1.07 (0.13,8.57) |  | 1.12 (0.67,1.89) |
|  |  |  |  |  |  |  |  |  |  |  |  |
| **CpG5 low** | 0.52 (0.13,2.11) |  | 3.16 (0.71,13.99) |  | 1.00 (0.37,2.73) |  | 0.51 (0.12,2.06) |  | - |  | 0.97 (0.43,2.20) |
| **CpG5 high** | 2.16 (1.09,4.31) |  | 2.92 (0.66,12.95) |  | 2.32 (1.20,4.47) |  | 1.88 (0.91,3.88) |  | 2.37 (0.30,18.99) |  | 1.94 (1.10,3.44) |
|  |  |  |  |  |  |  |  |  |  |  |  |
| **CpGs 1-5 low** | 0.96 (0.46,2.02) |  | 1.10 (0.22,5.47) |  | 0.88 (0.42,1.83) |  | 0.81 (0.39,1.67) |  | - |  | 0.87 (0.49,1.54) |
| **CpGs 1-5 high** | 1.30 (0.83,2.03) |  | 1.21 (0.43,3.39) |  | 1.25 (0.82,1.93) |  | 0.98 (0.63,1.51) |  | 1.01 (0.27,3.74) |  | 1.04 (0.74,1.48) |

**Table S18.** Bi-variate association between *NR3C1* methylation levels at baseline and duration of substance use (per year increase) assessed over three years in female participants of the KUPOL study, Sweden.

**Abbreviations** RR risk ratio, CI confidence interval. Methylation level 0% was considered as the reference category. Low methylation group: methylation levels below the median of detectable methylation and high methylation group: methylation levels above the median.


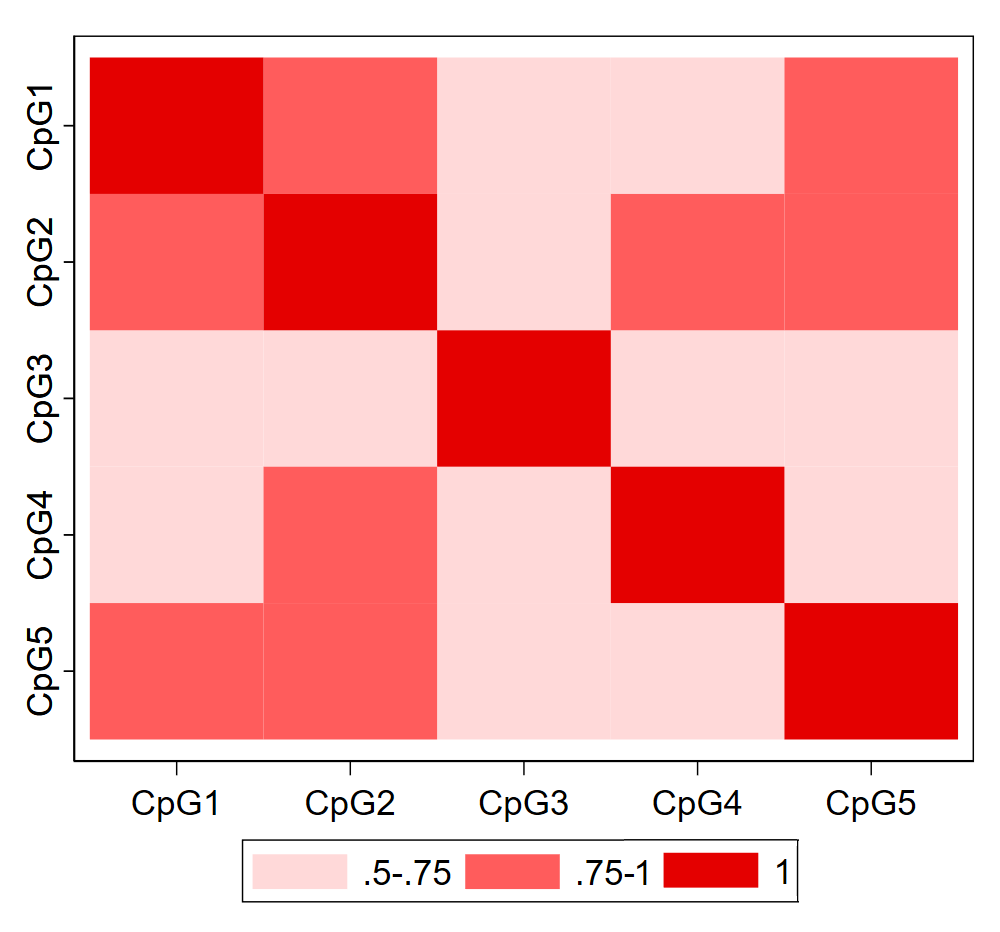


**Figure S1.** Correlation among the five 5 CpG sites under investigation. The red-color gradients correspond to the correlation strenght. Overall, the correlation among CpG sites ranged between a moderate (0.5-0.75) to strong positive (0.75-1) relationship. High correlations stemmed from the extremely right-skewed distribution of methylation levels for the five CpG sites and high prevalence of individuals with 0% of DNA methylated (from 49.5% to 91.5% of the sample).

**
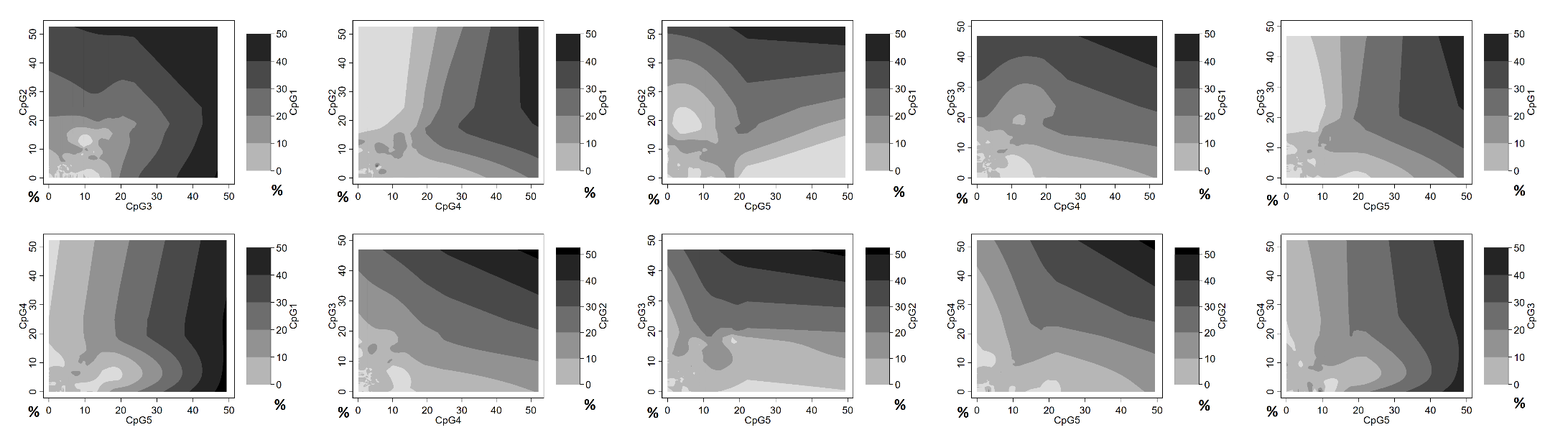
** **Figure S2.** Distribution of methylation levels of five CpG sites. These graphs visualize three-dimensional data in a two-dimensional plot. In particular, they show how the methylation level of a CpG site changes as a function of the levels of two other CpG sites on x and y axes (contour plot). Results support a moderate positive relationship among methylation levels at the five CpG sites.


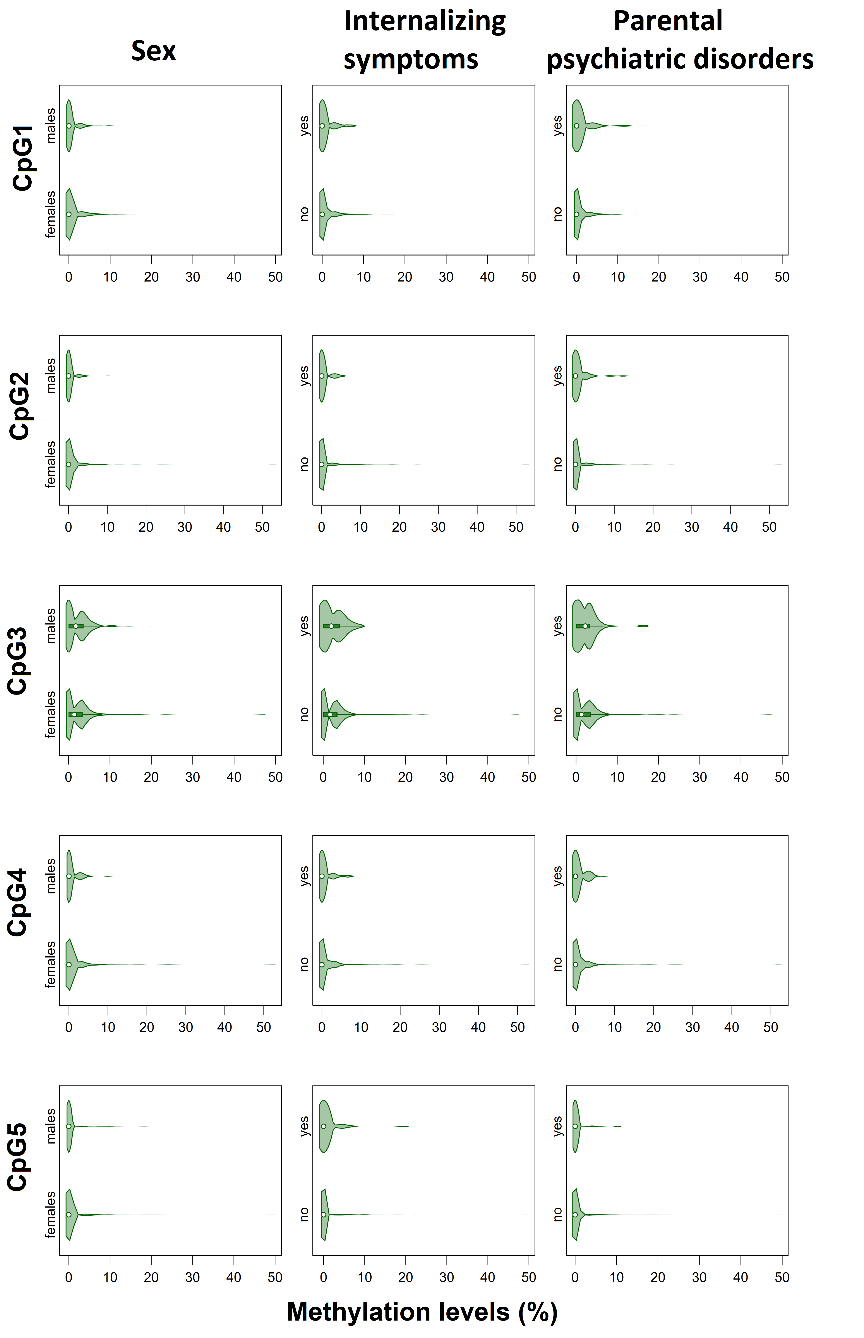


**Figure S3**. Distribution of methylation levels across the five *NR3C1* CpG sites in relation to sex, internalizing symptoms, and a parental history of psychiatric (i.e., mental, behavioral, and neurodevelopmental) disorders. The graphical visualization showed a relation between parental history of psychiatric disorders and higher levels of methylation at CpG sites 1, 2 and 3.


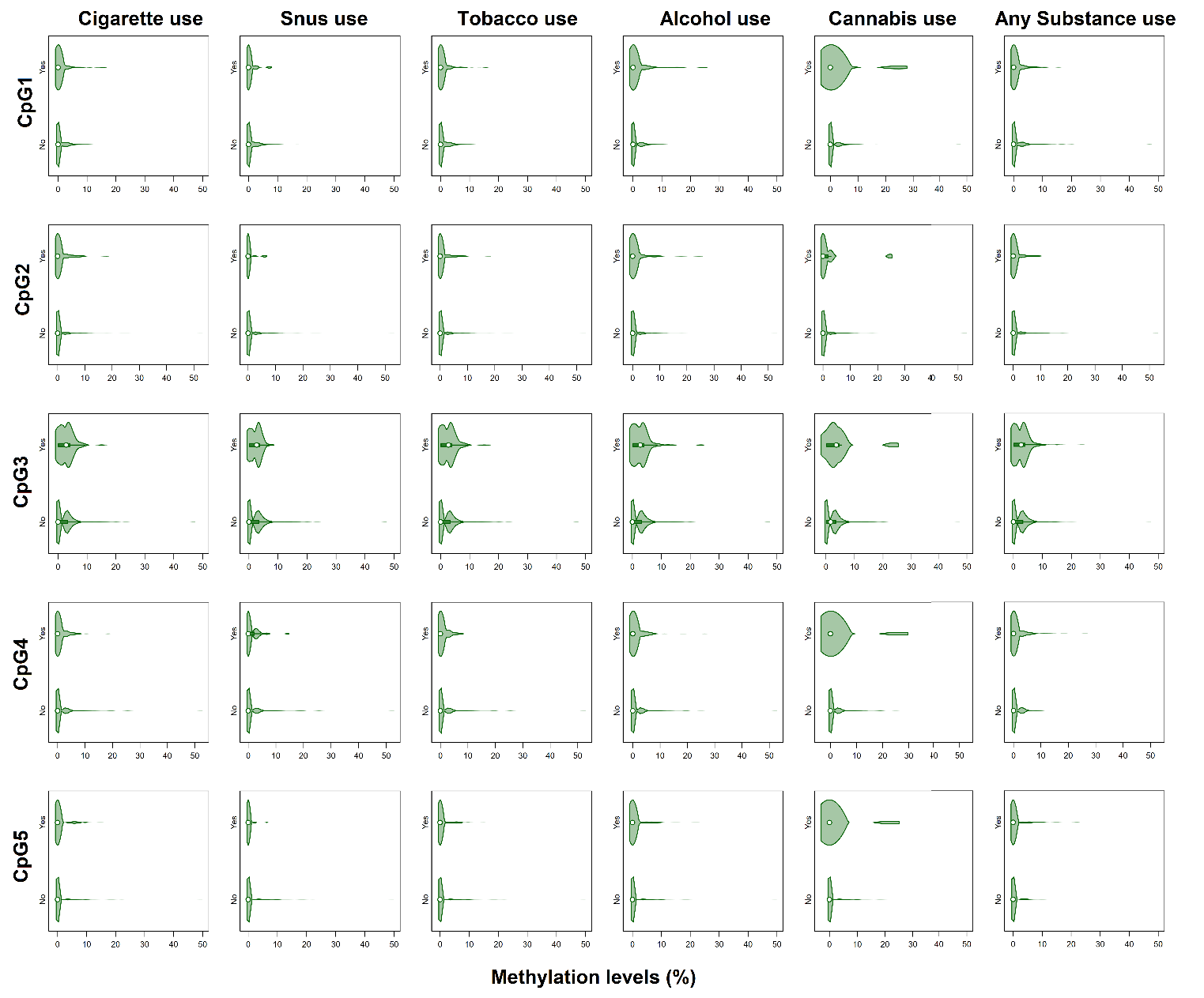


**Figure S4**. Distribution of methylation levels across the five NR3C1 CpG sites for recent substance use. The graphical visualization of the distribution of methylation levels supports higher methylation levels at CpG site 3 in relation to recent substance use.
